# Preventing *Salmonella* Choleraesuis infection by *Mume Fructus-Schisandra* formulas through the “membrane damage-virulence inhibition-immunomodulation” pathway

**DOI:** 10.3389/fmicb.2026.1781674

**Published:** 2026-04-14

**Authors:** Hua Chang, Manxia Chang, Bofang Duan, Yihan Wang, Minghao Peng, Shengjie Ren, Gang Duan, Xun Xiang

**Affiliations:** 1 College of Veterinary Medicine, Yunnan Agricultural University, Kunming, China; 2 Yunnan Center for Animal Disease Prevention and Control, Kunming, China; 3 College of Animal Science and Technology, Hunan Biological and Electromechanical Polytechnic, Changsha, China

**Keywords:** membrane damage, *Mume Fructus*, *Salmonella* Choleraesuis, *Schisandrae Chinensis Fructus*, virulence inhibition

## Abstract

**Introduction:**

The strong pathogenicity and environmental adaptability of *Salmonella* Choleraesuis cause significant losses to the pig breeding industry, and existing prevention and control strategies (such as the abuse of antibiotics and limitations in vaccine development) are urgently needed for optimization. This study aims to screen green prescriptions against *S. choleraesuis* from Chinese herbal medicines that possess both medicinal and edible value, providing a basis for the development of green feed additives.

**Methods:**

In this study, 10 Chinese herbal medicines from the *Catalog of Feed Raw Materials* were screened for antibacterial activity and synergistic compatibility via microbroth and microcheckerboard dilution assays. The minimum inhibitory concentration (MIC) and minimum bactericidal concentration (MBC) of the prescription against *S. choleraesuis* were determined. And the antibacterial activity was detected by extracellular potassium ion concentration, total leakage rate, transmission electron microscopy, and RT-qPCR analyses.

**Results:**

The optimal compound composition was determined via L9 (3^4^) orthogonal tests as follows: *Mume Fructus*, 30 g; *Schisandrae Chinensis Fructus*, 10 g; *Crataegi Fructus*, 5 g; and *Atractylodes lancea*, 5 g. Its MIC and MBC against *S. choleraesuis* were 15.625 mg/mL and 31.25 mg/mL, respectively. Furthermore, extracellular potassium ion concentration, total leakage rate, transmission electron microscopy, and RT-qPCR analyses demonstrated that the compound increased bacterial cell membrane permeability, disrupted bacterial flagella, detached cell membranes from cell walls, induced irregular vacuole formation, and severely impaired the structural integrity of certain bacterial cells. In addition, compound prescription inhibited the transcription levels of key genes in the type III secretion system (T3SS) (sipA/B/C, invA/F, prgH/I, spaP, hilA/D, *p* < 0.01). A mouse diarrhea model was established with 5 × 10^4^ CFU/mL *S. choleraesuis*. After 7 days of treatment (10 g/kg), the protection rate reached 66.7%. It significantly increased sIgA in the mice and downregulated the expression of inflammatory factors (TNF-α, IL-1β, and IL-6; *p* < 0.01).

**Conclusion:**

The screened Chinese herbal prescription has significant antibacterial activity against *S. choleraesuis* both *in vitro* and *in vivo*. The prescription acts against *S. choleraesuis* through a multitarget mechanism of “membrane damage-virulence inhibition-immune regulation,” which provides theoretical support for the prevention of *S. choleraesuis* and the development of green feed additives.

## Introduction

*Salmonellosis* is a global zoonotic disease that typically presents with symptoms of diarrhea, abdominal pain, fever, and vomiting and is a collective term for illnesses caused by bacteria of the genus Salmonella (Zhou et al., 2025; Chen et al., 2024). Salmonella can colonize the digestive tract of animals, is excreted in feces and spreads into the environment (Elbediwi et al., 2020; Wu et al., 2021). In affected pig herds, *Salmonella* Typhimurium is the prevalent strain, whereas *Salmonella* Choleraesuis is the main pathogenic serotype in piglets. In the global livestock production system, *Salmonella* Choleraesuis has emerged as a significant pathogen that poses a threat to pig health. It not only causes septicemia and refractory diarrhea in piglets, with a mortality rate of up to 90%, but also coinfects piglets with classical swine fever virus, resulting in significant economic losses in the Chinese pig farming industry. Owing to its strong environmental adaptability, this bacterium poses a high transmission risk across multiple stages, including breeding and processing, which severely restricts the sustainable development of the pig industry (Peeters et al., 2020; Chiu et al., 2006; Jajere, 2019).

In-depth studies on the pathogenic mechanisms of *Salmonella* Choleraesuis have revealed that its invasion process relies on virulence factors, including flagella, fimbriae, and the type III secretion system (T3SS). After oral ingestion, bacteria activate acid resistance mechanisms, migrate to the small intestine via flagella to invade M cells, adhere to intestinal epithelial cells through fimbriae, and form Salmonella-containing vacuoles (SCVs) via the type 3 secretion system (T3SS) encoded by Salmonella pathogenicity island 1 (SPI-1). The T3SS encoded by Salmonella pathogenicity island 2 (SPI-2) subsequently promotes bacterial proliferation within macrophages, ultimately leading to systemic infection. Among these factors, the T3SS serves as a key pathogenic apparatus, presenting enormous challenges for prevention and control due to the complexity of its structure and functional mechanisms. hilA is the core transcription factor of the SPI-1 T3SS, and hilD is the upstream regulatory gene of hilA. Both are master switches for the expression of all SPI-1 T3SS genes, and their inhibition can directly block the entire T3SS expression system. invA and invF encode the structural proteins of the T3SS needle complex, prgH encodes the inner membrane ring protein of the T3SS secretion apparatus, and spaP encodes the outer membrane protein of the secretion apparatus. These genes are essential for the structural integrity and secretion function of the T3SS. Effector protein genes (sipA, sipB, and sipC) encode the key effector proteins secreted by the T3SS. sipA promotes the rearrangement of host cell actin, sipB mediates the fusion of bacterial vacuoles and host cell membranes, and sipC forms actin filaments. These proteins are key for Salmonella invasion of host cells and cause cytotoxicity (Yip and Strynadka, 2006; Schraidt and Marlovits, 2011; Ellermeier and Slauch, 2007; Koraimann, 2003; Kauppi et al., 2007; Fahlen et al., 2001; Waterman and Holden, 2003). Currently, prevention and control strategies for *Salmonella* Choleraesuis primarily focus on managing the breeding environment, but transmission issues remain unresolved. Vaccine development has been hindered by serotype specificity and limitations in carrier clearance, while long-term misuse of antibiotics has led to increasingly severe drug resistance problems (Grigar et al., 2017). *Salmonella* can acquire drug resistance through changes in metabolic targets or inactivation of enzyme synthesis (Wang et al., 2024; Tang et al., 2022; Jiang et al., 2019). Since China implemented a complete ban on antibiotic growth promoters (AGPs) in animal feed in 2020, the search for green, safe, and efficient antibiotic substitutes has become an urgent priority for the industry.

Against this backdrop, traditional Chinese medicine (TCM) has become a research hotspot in anti-Salmonella studies due to its unique advantages, including being natural, residue-free, multitarget antibacterial, and less likely to induce drug resistance. The antimicrobial activity of single herbs, such as *Schisandrae Chinensis Fructus* and *Atractylodis Rhizoma*, as well as the antibacterial effects achieved by TCM compound formulas through component combinations, highlights the significant potential of TCMs in preventing and controlling Salmonella. However, current research on the mechanisms of TCM compound formulas against *Salmonella* Choleraesuis is still insufficient, and their antibacterial efficacy, both *in vitro* and *in vivo*, as well as their action targets, need to be further clarified.

On the basis of this foundation, this study selected 10 TCM herbs from the “raw material catalog” of medicinal and edible homologous plants. *Lablab album semen* (white hyacinth bean) has significant antibacterial and antiviral activities. *Atractylodis Rhizoma* (attractylodes) has antitumor, anti-inflammatory, and antibacterial effects, as well as liver protection. *Magnoliae officinalis Cortex* (M. bark) has antibacterial and antioxidant effects, promotes gastrointestinal motility, and alleviates abdominal distension and diarrhea. *Lophatheri Herba* (bamboo leaves) exhibit heat and has antibacterial and anti-inflammatory effects. *Crataegi Fructus* (hawthorn) aids in digestion, offers antibacterial properties, and provides cardiovascular protection. *Mume Fructus* (smoked plum) has antibacterial effects, regulates immunity, and can be used to treat ulcerative colitis. *Schisandrae chinensis Fructus* (schisandra) offers liver protection and has antioxidant, antibacterial, and anti-inflammatory effects. *Lonicerae* japonica flos (honeysuckle) has antibacterial and antiviral effects and dispels wind heat. *Pogostemonis Herba* (patchouli) regulates digestion and resists pathogenic microorganisms. *Psoraleae Fructus* (Psoralea) warms the kidneys to prevent diarrhea and has antibacterial, anti-inflammatory, and antitumor effects (Guo et al., 2015; Pu et al., 2021; Jiang et al., 2013).

This study first evaluated the antibacterial effects of these individual herbal medicines and then optimized the formula through *in vitro* antibacterial tests and orthogonal experiments. This study systematically explored the effects of these compounds on the cell membrane permeability, ultrastructure, and T3SS gene expression of *Salmonella* Choleraesuis and evaluated their *in vivo* efficacy in a mouse diarrhea model. This research aims to provide a scientific basis for the development of green feed additives and establish novel approaches for the prevention and control of *Salmonella* Choleraesuis. These findings hold significant theoretical and practical implications for advancing the green and sustainable development of the pig farming industry.

## Experimental materials

In this study, the Chinese herbal medicines listed in the *Raw Material Catalog* of Announcement issued by the Ministry of Agriculture and Rural Affairs of China, including *Lablab album semen*, *Atractylodes lancea*, *Magnolia officinalis*, *Lophatherum gracile*, *Crataegus pinnatifida*, *Mume Fructus*, *Schisandra chinensis*, *Lonicera japonica*, *Psoralea corylifolia*, and *Pogostemon cablin,* were selected. These medicinal materials were purchased from the traditional Chinese medicine market in Kunming, Yunnan Province, in October 2023. The tested strain, *Salmonella* Choleraesuis, was isolated and preserved in the laboratory of Yunnan Agricultural University. Six- to eight-week-old ICR mice weighing 18–22 g, with equal numbers of males and females, were purchased from the Experimental Animal Center of Yunnan University. The mice were adaptively fed for 3 days before the experiment, with daily water replacement and free access to food (Taddeo et al., 2024).

## Screening of single Chinese medicines against *Salmonella* Choleraesuis

The ten Chinese medicines were crushed and sieved. The water extracts (1 g/mL of crude drug) were prepared via decoction, filtration, centrifugation, and concentration. After resuspension, *Salmonella* Choleraesuis was streaked on plates, cultured in liquid medium, and diluted to 0.5 McFarland turbidity for use.

The Oxford cup assay was performed by spreading the bacteria on MHA plates, adding single-drug solutions, and measuring the diameter of the inhibition zone after incubation. Sensitivity was determined according to the standards of Pharmacology of Chinese Materia Medica [highly sensitive (inhibition zone ≥20 mm), moderately sensitive (15–19 mm), slightly sensitive (10–14 mm), and resistant (<10 mm)]. The minimum inhibitory concentration (MIC) and minimum bactericidal concentration (MBC) were determined via the microbroth dilution method. Gradient concentrations of drug solutions and bacterial solutions were set in 96-well plates, and the concentrations were determined by observing turbidity and colony growth.

On the basis of the antibacterial sensitivity results, single drugs with superior inhibitory effects were selected for checkerboard microdilution assays to evaluate their combined antibacterial activity. The fractional inhibitory concentration (FIC) index was calculated to determine drug interactions [synergy (FIC < 0.5), additive effect (FIC 0.5–1), indifference (FIC 1–2), or antagonism (FIC > 2)].

## Screening of compound Chinese medicines against *Salmonella* Choleraesuis

On the basis of the FIC results of single herbs, antibacterial effects were selected, and 8 groups of compound combinations were designed according to the orthogonal array. The corresponding Chinese medicines were prepared through crushing, soaking, decocting, filtering, centrifuging, concentrating, and uniformly formulation at a concentration of 1 g/mL (crude drug amount).

A total of 100 μL of *Salmonella* Choleraesuis bacterial mixture diluted to 0.5 McFarland turbidity was taken and evenly spread on an MHA plate. After the bacterial mixture was absorbed, Oxford cups were placed vertically on the plate surface, and 100 μL of each compound mixture was added. The plates were then incubated in a 37 °C constant-temperature incubator for 18–24 h. After incubation, the diameter of the inhibition zone was measured with a Vernier caliper, with each group measured in triplicate, and the average value was recorded. Finally, the range analysis method was employed to analyze the test results systematically and screen out four Chinese medicinal herbs with strong inhibitory effects.

Four Chinese medicinal herbs were selected to design an L9 (3^4^) orthogonal array with three dosage levels (Tables 1, 2). In accordance with the orthogonal array, 9 kinds of Chinese medicines were prepared, and the inhibition zone diameter of each formula was measured via the Oxford cup method, with each group measured in triplicate. The test results were analyzed via the range method to identify the optimal dosage combination with good anti-*Salmonella* Choleraesuis effects, specifically to determine the effectiveness of the Chinese medicine.

**Table 1:** L_9_ (3^4^) orthogonal table.

Number	Factors			
	A	B	C	D
1	1	1	1	1
2	1	2	2	2
3	1	3	3	3
4	2	1	2	3
5	2	2	3	1
6	2	3	1	2
7	3	1	3	2
8	3	2	1	3
9	3	3	2	1

Factors A, B, C, and D correspond to *Mume Fructus, Schisandra chinensis, Crataegi Fructus*, and *Atractylodis Rhizoma*, respectively.

**Table 2:** Herbal dose level settings.

Factors	A	B	C	D
Level 1	5 g	5 g	5 g	5 g
Level 2	20 g	10 g	10 g	10 g
Level 3	30 g	20 g	20 g	20 g

Factors A, B, C, and D correspond to *Mume Fructus*, *Schisandra chinensis*, *Crataegi Fructus*, and *Atractylodis Rhizoma*, respectively.

## Determination of the MIC and MBC of the screened Chinese herbal formulation against *Salmonella* Choleraesuis

The four Chinese herbs were accurately weighed, mixed, and then soaked in water at a 1:10 ratio for 30 min. The mixture was decocted in a 60 °C water bath for 25 min and filtered. The mixture was subsequently centrifuged at 5,000 rpm for 5 min, after which the supernatant was collected. The mixture was then concentrated under negative pressure at 58 °C. The concentrate was dried to obtain an extract, which was subsequently dissolved, and a compound mixture with a concentration of 1 g/mL (equivalent to the raw herb) was prepared. The mixture was sterilized at 121 °C for 15 min and stored at 4 °C.

The compound solution was diluted to gradient concentrations of 1,000, 500, 250, 125, 62.5, 31.25, 15.625, 7.9125, and 3.90625 mg/mL. In accordance with CLSI (2020) guidelines, 0.5 McFarland standard bacterial suspensions were diluted to 1 × 10^5^ CFU/mL. A microbroth dilution assay was performed in a 96-well plate. After inoculation, the mixture was incubated at 37 °C for 18–24 h, and turbidity and bacterial growth were observed. The MIC and MBC values were then determined.

## Network pharmacology analysis of the optimal traditional Chinese medicine for the treatment of *Salmonella* Choleraesuis

After screening the optimal formula for antibacterial activity, protein–protein interaction analysis in network pharmacology was conducted on the basis of its active components and corresponding targets. A hub network diagram and drug-component-target network diagram were constructed via Cytoscape 3.10.3 software. The DAVID database was used to perform GO functional enrichment analysis and KEGG pathway enrichment analysis on the overlapping inflammatory targets via the SRPLOT platform. Key target proteins in the Hubbs network were selected as receptor proteins (retrieved from RCSB PDB^1^), and molecular docking was performed via the CD-DOCK2 platform to verify the possibility of interaction between the target proteins and ligands (active components of the drug) (Hong et al., 2023; Huang et al., 2024).

## Study of the antibacterial mechanism of the optimal traditional Chinese medicine prescription against *Salmonella* Choleraesuis

### Effect on bacterial cell membrane permeability

The degree of damage to the cell membrane was evaluated by measuring the OD₆₀₀ value and the extracellular ion concentration. *Salmonella* Choleraesuis was activated to the logarithmic growth phase (OD = 0.6) and then divided into four groups. The compound mixture was added to each group at final concentrations of 1/4 MIC, 1/2 MIC, and MIC, with the bacterial mixture used as the control. The cultures were incubated at 37 °C with shaking at 140 r/min. Samples were taken at 4, 10, and 18 h. The K^+^ content in the supernatant was determined via a potassium ion concentration colorimetric kit, and the absorbance at 600 nm was measured to evaluate the total leakage rate. Each group was tested 3 times, and two-way analysis of variance and graphing were performed via GraphPad Prism 9.5.0.

### Effect on bacterial morphology

Bacterial solutions from the 1/4 MIC, 1/2 MIC, and MIC concentration groups, as well as the blank group, were collected at 2, 6, and 8 h, respectively. After centrifugation at 12,000 rpm for 5 min, the bacteria were fixed with 0.5% glutaraldehyde. The effects of traditional Chinese medicine on the ultrastructure of bacterial cells were observed via transmission electron microscopy.

### Effect on the transcriptional levels of key genes in the T3SS system

RT-qPCR primers for key T3SS genes (*sipA*, *sipB*, *sipC*, *hilA*, *hilD*, *invA*, *invF*, *spaP*, *prgH*) and the internal reference gene 16SrDNA (Table 3) were designed on the basis of GenBank gene sequences.

**Table 3:** Fluorescence quantitative primer information.

Primer name	5′-3’ Primer sequences	Product/bp
*sipA*	F: GCGTAACCAGCAAGAGCATT	171
	R: CCGTCGTGTCTGATTGTAAGG	
*sipB*	F: CTGATTGGCAAGGCGATTACC	106
	R: TGGCAATAGCGGCGACAA	
*sipC*	F: CACGACTAAAGCGAATGAGGTT	101
	R: CAACGGCACTGGAAGACATT	
*hilA*	F: CTCATACATTGGCGATACTTCCTT	143
	R: GCGGCAGTTCTTCGTAATGG	
*hilD*	F: GCCAGAAGAGAGGTATTTGAACA	142
	R: CAGTAAGCAGGAACAGCAGAA	
*invA*	F: TGTTCGTCATTCCATTACCTACC	207
	R: CGGCATCGGCTTCAATCAA	
*invF*	F: AGCGGCAACACGATGAGAA	203
	R: TGTGAAGGCGATGAGTAACCA	
*spaP*	F: AGTATGGAGAAGAGACCGAGAC	148
	R: ACGACAACAAAGGGCAAATAGA	
	R: CTGAATAATGGCAGCATCAATATCC	
*prgH*	F: ACAGGTCGGTGAATTGCTTATC	194
	R: GCTGCGGCGAGTTAAGTATC	
*16S rDNA*	F: TTGTTGCCAGCGATTAGGTC	232
	R: CGATTACTAGCGATTCCGACTT	

The RNA was extracted using the TRIzol method. One milliliter of bacterial culture was lysed with TRIzol, and the RNA was precipitated with isopropanol and washed twice with 1 mL of 75% ethanol. After the ethanol evaporated, the RNA was dissolved in RNase-free water and stored at −80 °C. Finally, the concentration and purity of the RNA were detected via a nucleic acid microspectrophotometer. Using the extracted RNA as a template, reverse transcription was performed according to the reaction system and procedure shown in Table 4.

**Table 4:** RNA reverse transcription reaction system.

Reagents	Volum (μL)	RNA reverse transcription procedure	
		Temperature	Time
2xTS reaction mix	10	25 °C	10 min
Random primer	1	42 °C	30 min
		85 °C	5 s
gDNA remover	1	16 °C	Forever
Enzyme mix	1		
Total RNA	1		
RNase-free water	6		
Total volume	20		

The SYBR® Premix Ex Taq™ kit was used to detect the expression levels of key genes in T3SS (*sipA*, *sipB*, *sipC*, *hilA*, *hilD*, *invA*, *invF*, *spaP*, *prgH*) and the 16S RNA gene following the fluorescent PCR system and procedure. After the experiment, the CT values were statistically analyzed using the 2^-ΔΔT^ method. The graphs were plotted via GraphPad Prism 9.5.0.

## Establishment of a mouse diarrhea model induced by *Salmonella* Choleraesuis

*For the animal regression test*, eight 6-8-week-old ICR mice (weighing 18–22 g, half male and half female) were selected and randomly divided into 2 groups. The experimental group was intraperitoneally injected with a bacterial mixture, whereas the control group received normal saline injections. The dead mice were dissected, and the diseased tissues were subjected to enrichment culture. Isolation was performed via MacConkey agar media, and the strains were identified through a combination of biochemical tests, Gram staining, and PCR sequencing.

The optimal bacterial challenge concentration was as follows: *Salmonella* Choleraesuis was cultured in NA liquid medium (37 °C, 140 r/min, 18 h) and then diluted to bacterial suspensions of 5 × 10^5^, 1 × 10^5^, 5 × 10^4^, and 1 × 10^4^ CFU/mL. Each mouse was intraperitoneally injected with 100 μL of the bacterial suspension. Five groups of mice (four mice in each group) were adaptively fed for 3 days, fasted for 12 h, and subsequently injected with different concentrations of the substance. The control group was injected with normal saline. The diarrhea and mortality rates were determined to determine the optimal modeling concentration. All the mice were housed in individually ventilated cages at the Laboratory Animal Center of Yunnan Agricultural University, with the room temperature controlled at 22 ± 2 °C, a relative humidity of 50–60%, a 12-h light/12-h dark cycle, sterilized bedding, and ad libitum access to standard sterilized chow and water.

## Evaluation of the efficacy of traditional Chinese medicine prescriptions against *Salmonella* Choleraesuis

A bacterial suspension was prepared at the selected concentration, and the TCM was formulated at a concentration of 1 g/mL. Sixty mice (divided into 6 groups with 10 mice in each group) were adaptively fed, the bacterial groups (BG) were injected with bacterial suspensions, the control group (CG) was injected with normal saline, and the antibiotic group (AG) was given enrofloxacin (10 g/kg). The traditional Chinese medicine (TCM) groups were treated with the formulated drug after being infected with bacteria to induce morbidity and were given 10 g/kg TCM.

The mice were recorded daily, and the protection rate was calculated. The jejunum tissue was collected to detect *sIgA* via ELISA, and the data were analyzed via one-way analysis of variance. Primers for *TNF-α*, *IL-1β*, and *IL-6* were designed (Table 5). Total blood RNA was extracted via TRIzol, and fluorescent quantitative PCR was performed via the SYBR Premix Ex Taq kit. The 2^-ΔΔT^ method was used for data analysis, and the graphs were plotted via GraphPad Prism 9.5.0. The jejunal tissues were subjected to pathological section analysis to observe the changes in intestinal tissues after the administration of the TCM.

**Table 5:** Primer sequences for fluorescence quantification.

Primer name	5′-3’ Primer sequences	Product/bp
*TNF-α*	F: TCAGAATGAGGCTGGATAAGATC	294
	R: AAGAGGAGGCAACAAGGTAGA	
*IL-1β*	F: GGCAGGCAGTATCACTCATTG	162
	R: CCAGCAGGTTATCATCATCATCC	
*IL-6*	F: TTCCATCCAGTTGCCTTCTTG	141
	R: AATTAAGCCTCCGACTTGTGAA	
*GAPDH*	F: CGACCCCTTCATTGACC	198
	R: CCAGTAGACTCCACGACA	

## Data analysis

GraphPad Prism 9.5.0 software was used for data visualization and statistical analysis, and the results are expressed as the means ± standard errors (means ± SEs). Data with a normal distribution and homogeneous variance were analyzed with *post hoc* comparisons via Tukey’s method. The significance of differences was determined as *p* > 0.05 (no significant difference), *p* < 0.05 (a statistically significant difference existed), and *p* < 0.01 (a statistically significant difference existed).

## Results

### Screening of Chinese herbal medicines against *Salmonella* Choleraesuis

The results showed that *Mume Fructus*, *Schisandrae Chinensis Fructus*, and *Magnoliae officinalis* had better antibacterial effects, followed by *Crataegi Fructus*, *Atractylodis Rhizoma*, and *Agastaches Herba*, while *Lophatheri Herba* had poor antibacterial effects. *Schisandrae Chinensis Fructus* had the strongest antibacterial activity (MIC of 15.625 mg/mL, MBC of 31.25 mg/mL), followed by *Mume Fructus* and *Magnoliae officinalis* Cortex. The MIC of *Mume Fructus* was 31.25 mg/mL, the MBC was 62.5 mg/mL, and both the MIC and MBC of *Magnoliae officinalis* Cortex were 31.25 mg/mL. For *Crataegi Fructus*, the MIC was 125 mg/mL, and the MBC was 250 mg/mL. For *Atractylodis Rhizoma*, the MIC was 250 mg/mL, and the MBC was 500 mg/mL. For *Lophatheri Herba*, the MIC was 200 mg/mL, and the MBC was 400 mg/mL. *Lablab Semen Album*, *Lonicerae Japonicae Flos*, *Agastach Herba*, and *Psoraleae Fructus* showed no significant antibacterial activity (MIC/MBC ≥ 500 mg/mL). The results showed that 5 combinations exhibited synergistic effects. The nine formulations exhibited additive effects, and no antagonistic effects were observed when the microcheckerboard dilution method was used. The five formulations were as follows: *Fructus-Magnoliae Officinalis* Cortex (FIC = 0.625), *Schisandrae Chinensis Fructus-Magnoliae Officinalis Cortex* (FIC = 1), *Crataegi Fructus-Magnoliae Officinalis* Cortex (FIC = 0.75), *Schisandrae Chinensis Fructus-Atractylodis Rhizoma* (FICI = 0.75), and *Crataegi Fructus-Atractylodis Rhizoma* (FIC = 0.625).

### Screening of traditional chinese medicine prescriptions against *Salmonella* Choleraesuis

The test revealed that *Schisandra chinensis*, *Mume Fructus*, *Atractylodes lancea*, *Crataegus pinnatifida*, *Magnolia officinalis* and *Lophatherum gracile* were the main components of the prescription. The diameter of the inhibition zone was as follows: *Schisandra chinensis* > *Mume Fructus* > *Atractylodes lancea* > *Crataegus pinnatifida*. However, the R values for *Magnolia officinalis* and *Lophatherum gracile* were negative, indicating that *Schisandra chinensis*, *Mume Fructus*, *Atractylodes lancea*, and *Crataegus pinnatifida* were the primary components responsible for the antibacterial activity of the prescription against *Salmonella* Choleraesuis. The L9 (3^4^) orthogonal test was used to optimize the dosage, and the optimal combination was determined to be 30 g of *Mume Fructus*, 10 g of *Schisandra chinensis*, 5 g of *Crataegus pinnatifida* and 5 g of *Atractylodes lancea* (Table 6). The MIC of this is 15.625 mg/mL, and the MBC is 31.25 mg/mL, indicating good antibacterial effects.

**Table 6:** Preferred factors: Results of the antibacterial effect of each combination on Salmonella cholerae in pigs.

Trial number	Factor				Inhibition diameter (mm)
	A	B	C	D	
1	1	1	1	1	12.90
2	1	2	2	2	12.04
3	1	3	3	3	10.49
4	2	1	2	3	12.13
5	2	2	3	1	13.20
6	2	3	1	2	12.88
7	3	1	3	2	13.10
8	3	2	1	3	13.31
9	3	3	2	1	14.70
k1	11.81	12.71	13.03	13.60	*n* = 3
k2	12.74	12.85	12.96	12.67	
k3	13.70	12.69	12.26	11.98	
R	1.89	0.16	0.77	1.62	

Factors A, B, C, and D represent *Mume Fructus*, *Schisandra Chinensis*, *Crataegi Fructus*, and *Atractylodis Rhizoma*, respectively. k1: Mean value of level 1 for each factor; k2: Mean value of level 2 for each factor; k3: Mean value of level 3 for each factor; R: Range; *n*: Number of replicates per group.

### Acquisition of the optimal Chinese medicine formulation and inflammatory targets and the chinese medicine formulation-target-inflammation interaction network

**Figure 1:**
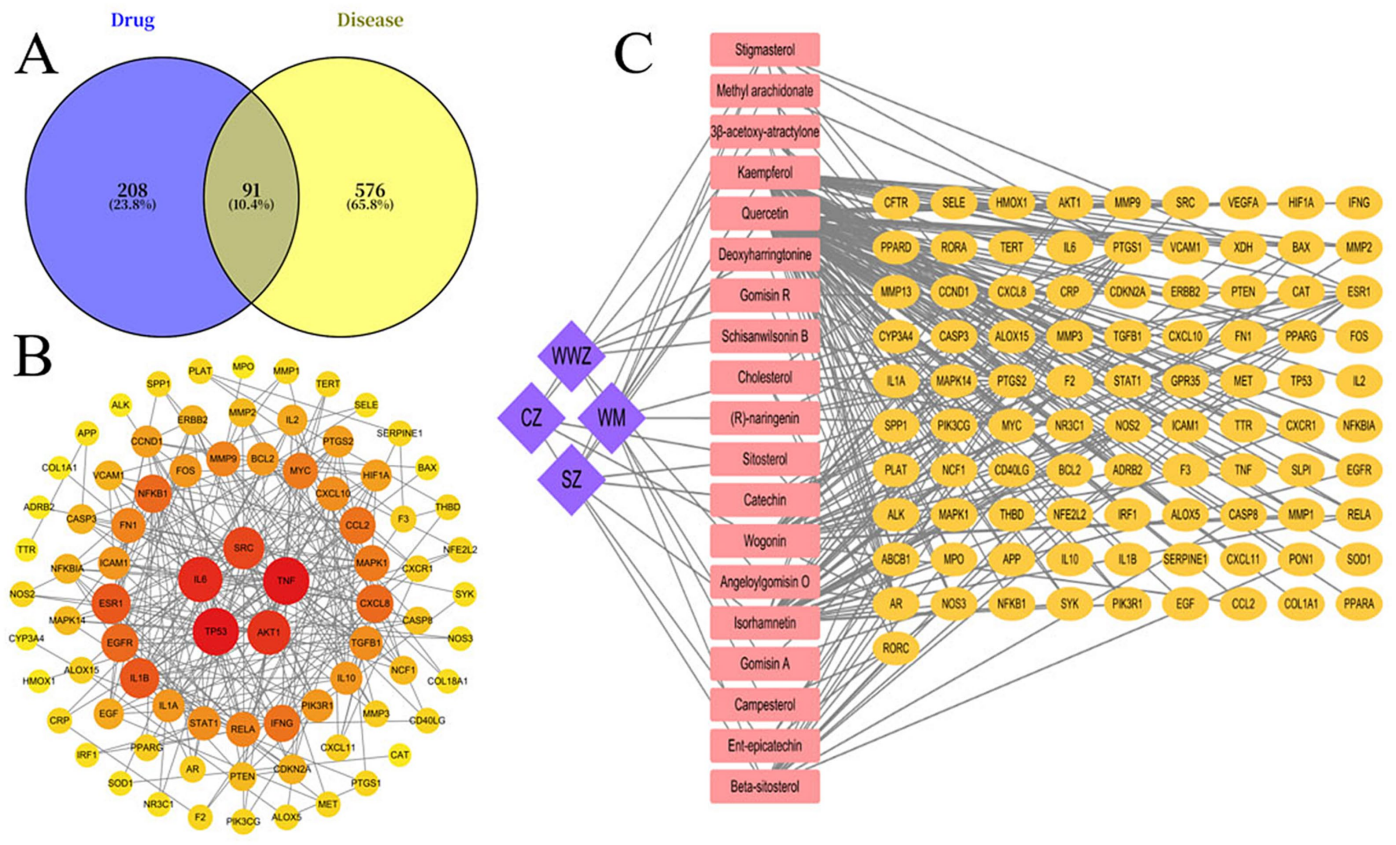
Protein–protein interaction network of potential anti-inflammatory targets for the traditional Chinese medicine formula: **(A)** Anti-inflammatory targets, **(B)** hub protein network of anti-inflammatory targets for the drug, **(C)** drug-component-target network diagram. In the diagram, a larger node size and darker color indicate a higher degree of importance of the node.

Analysis of the Hubbs network diagram revealed a total of 91 core targets related to inflammation for this formula, with the main targets including TP53, TNF, IL6, AKT1 and SRC (Figures 1A,B). Key target proteins (TNF and IL6) in the Hubbs network were selected as receptor proteins, and molecular docking was performed via the CD-DOCK2 platform to verify the possibility of interaction between the target proteins and ligands of active components of the drug (Table 7).

**Table 7:** Properties of the main core targets.

Target	Betweenness centrality	Closeness centrality	Degree
TP53	0.12777404780129656	0.45930232558139533	24
TNF	0.21000258683471015	0.5374149659863946	24
IL6	0.09780497035632574	0.4968553459119497	22
AKT1	0.151647866730053	0.4906832298136645	21
SRC	0.12275703783241687	0.4817073170731707	19

Through analysis of the drug-component-target network diagram, the main anti-inflammatory active components in the compound were screened out on the basis of the 91 core targets. Specifically, 8 active components were identified from *Mume Fructus*, corresponding to 88 anti-inflammatory targets. Five active components from *Schisandra chinensis were identified*, corresponding to 5 anti-inflammatory targets. Five active components from *Atractylodes lancea*, corresponding to 28 anti-inflammatory targets, and 7 active components from *Crataegus pinnatifida*, corresponding to 88 anti-inflammatory targets, were identified. Among these active components, *quercetin* had the highest degree of value. In terms of the degree values of the drugs, those of *Mume Fructus*, *Schisandra chinensis*, *Atractylodes lancea*, and *Crataegus pinnatifida* were 8, 5, 5, and 7, respectively, with *Mume Fructus* having the largest degree value (Figure 1C).

**Figure 2:**
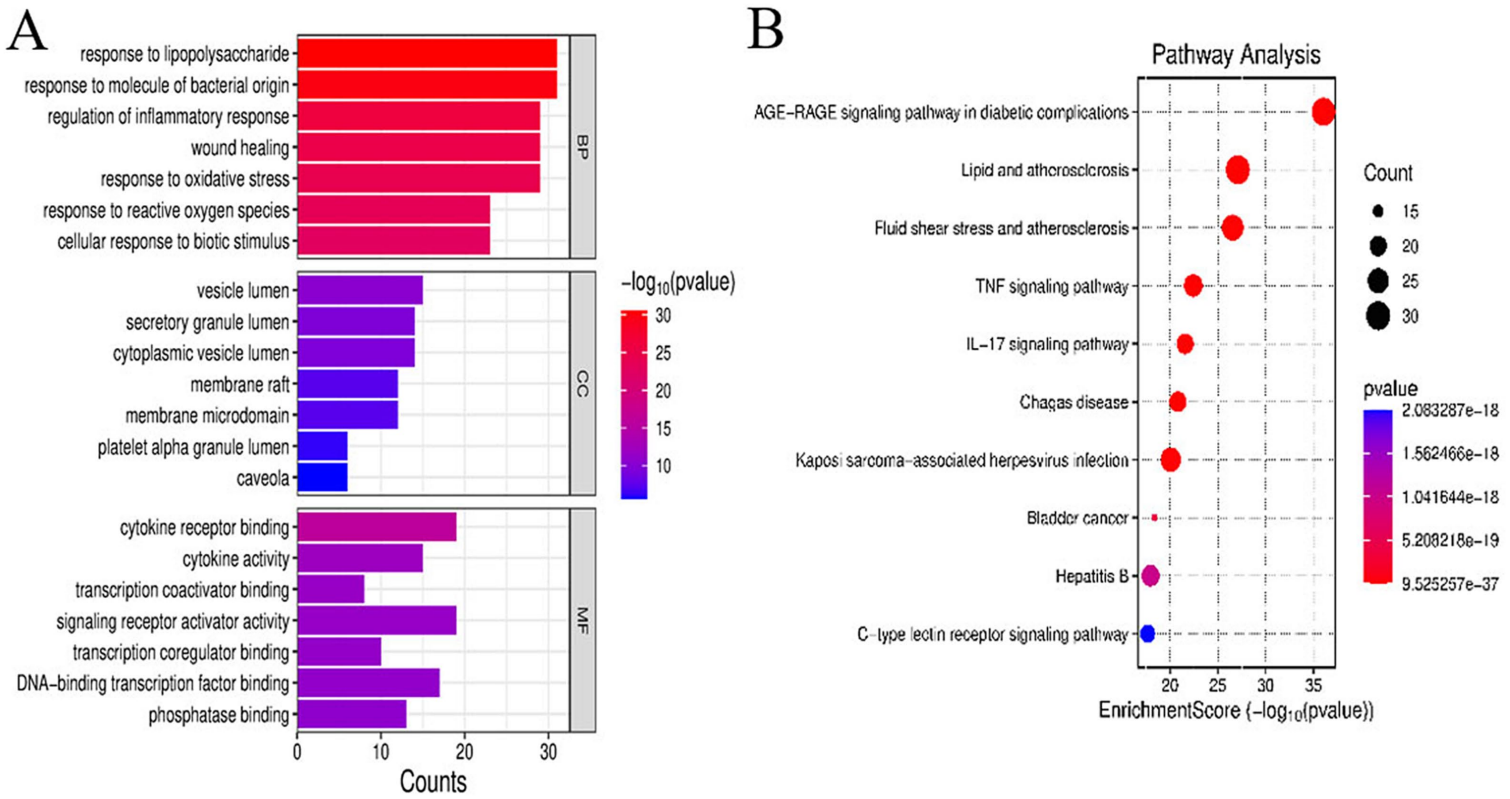
GO functional enrichment analysis and KEGG pathway enrichment analysis of core targets: **(A)** analysis of biological processes, cellular components, and molecular functions of core targets, **(B)** KEGG analysis of core targets. In the GO analysis chart, the ordinate represents the items of the GO analysis, and the abscissa represents the enrichment score. For biological processes (BP), cellular components (CC), and molecular functions (MF), the color change from blue to red indicates a change in the *p* value.

GO functional analysis revealed that the above-screened inflammation-related targets are involved in the following biological processes: response to lipopolysaccharides, response to bacteria-derived molecules (such as exotoxins, fimbrial proteins, flagellins, and metabolites), and regulation of the body’s inflammatory response. In terms of cellular components, these targets are localized mainly in the cytoplasm and cell membrane. The main molecular functions of these genes were cytokine receptor binding and cytokine activity (Figure 2A). As revealed by the KEGG pathway enrichment analysis, these targets are associated primarily with the TNF signaling pathway, which is involved in multiple aspects, including the inflammatory response (Figure 2B).

**Figure 3:**
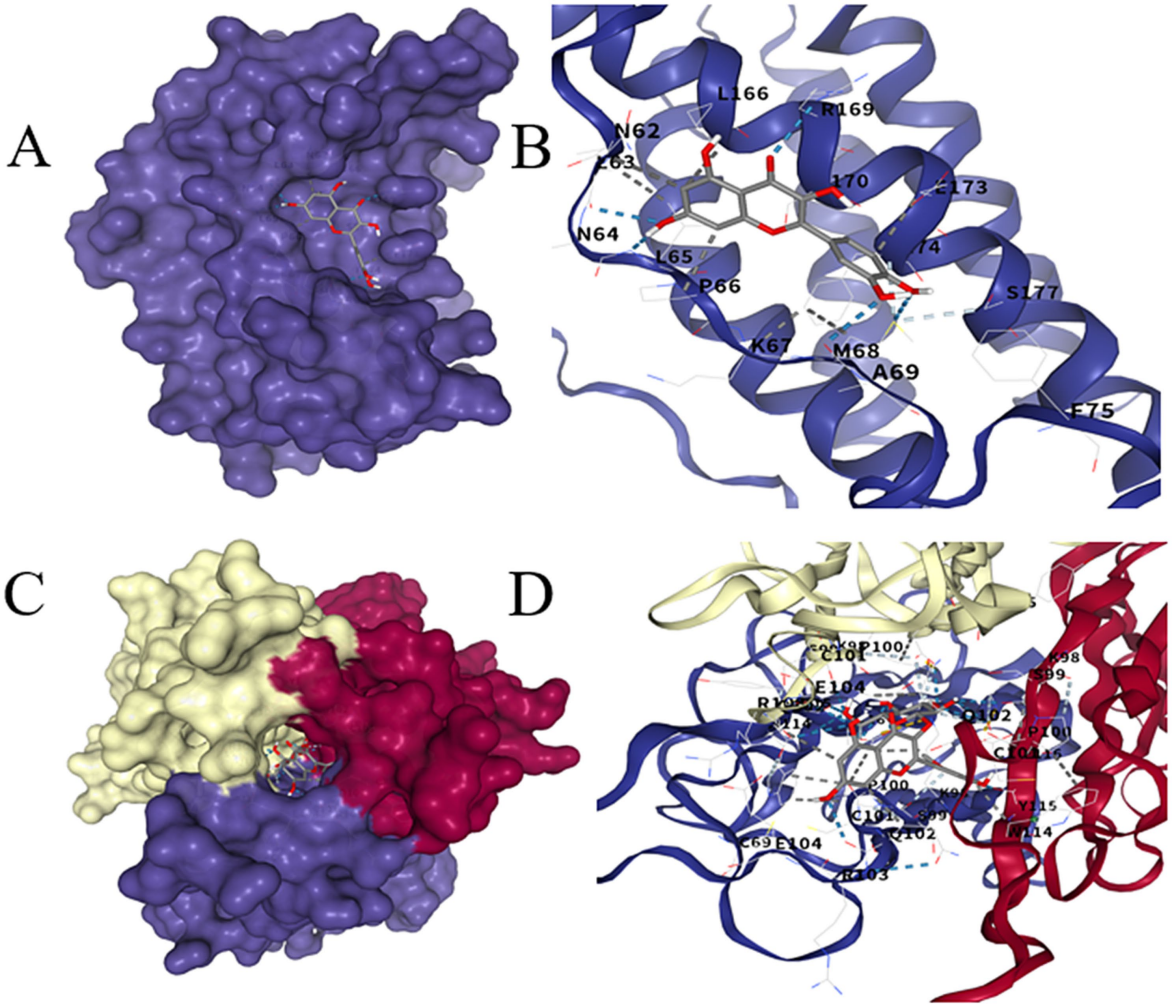
Docking diagrams of drugs with IL-6 and TNF-α. **(A)** Overall diagram of drug docking with IL-6. **(B)** Local interaction diagram of drug docking with IL-6. **(C)** Overall diagram of drug docking with TNF-α. **(D)** Local interaction diagram of drug docking with TNF-α.

Through analysis, the binding energies of quercetin and isorhamnetin (derived from Mume Fructus and Crataegi Fructus) to the targets IL-6 and TNF-α were obtained. IL-6 (PDB ID: 1IL6) showed the highest affinity for quercetin and isorhamnetin, with a binding energy of −7.2 kcal/mol for both (Figure 3A). TNF-α (PDB ID: 1TNF) exhibited the highest affinity for isorhamnetin, with a binding energy of −10.8 kcal/mol (Figure 3C). Quercetin forms relatively stable hydrogen bonds with the amino acid residues Asn:64, Leu:65, Met:68, and Arg:169 of IL-6 and hydrophobic bonds with the amino acid residues Asn:62, Pro:66, Leu:166, and Glu:173 of IL-6. Hydrogen bonds and hydrophobic bonds are the main forces dominating the binding of quercetin to IL-6 (Figure 3B).

Isorhamnetin establishes relatively stable hydrogen bonds with the amino acid residues of TNF-α, including Arg A:103, Tyr A:115, Glu A:116, Ser B:99, Tyr B:115, Glu B:116, Ser C:99, Pro C:100, Gln C:102, Arg C:103, and Glu C:116. Among these, 3 hydrogen bonds are formed with Glu A:116, and 2 hydrogen bonds are formed with Glu B:116 and Gln C:102. Hydrogen bonds are the main force dominating the binding of isorhamnetin to TNF-α (Figure 3D).

### *In vitro* investigation of the virulence of *Salmonella* Choleraesuis via traditional Chinese medicine

**Figure 4:**
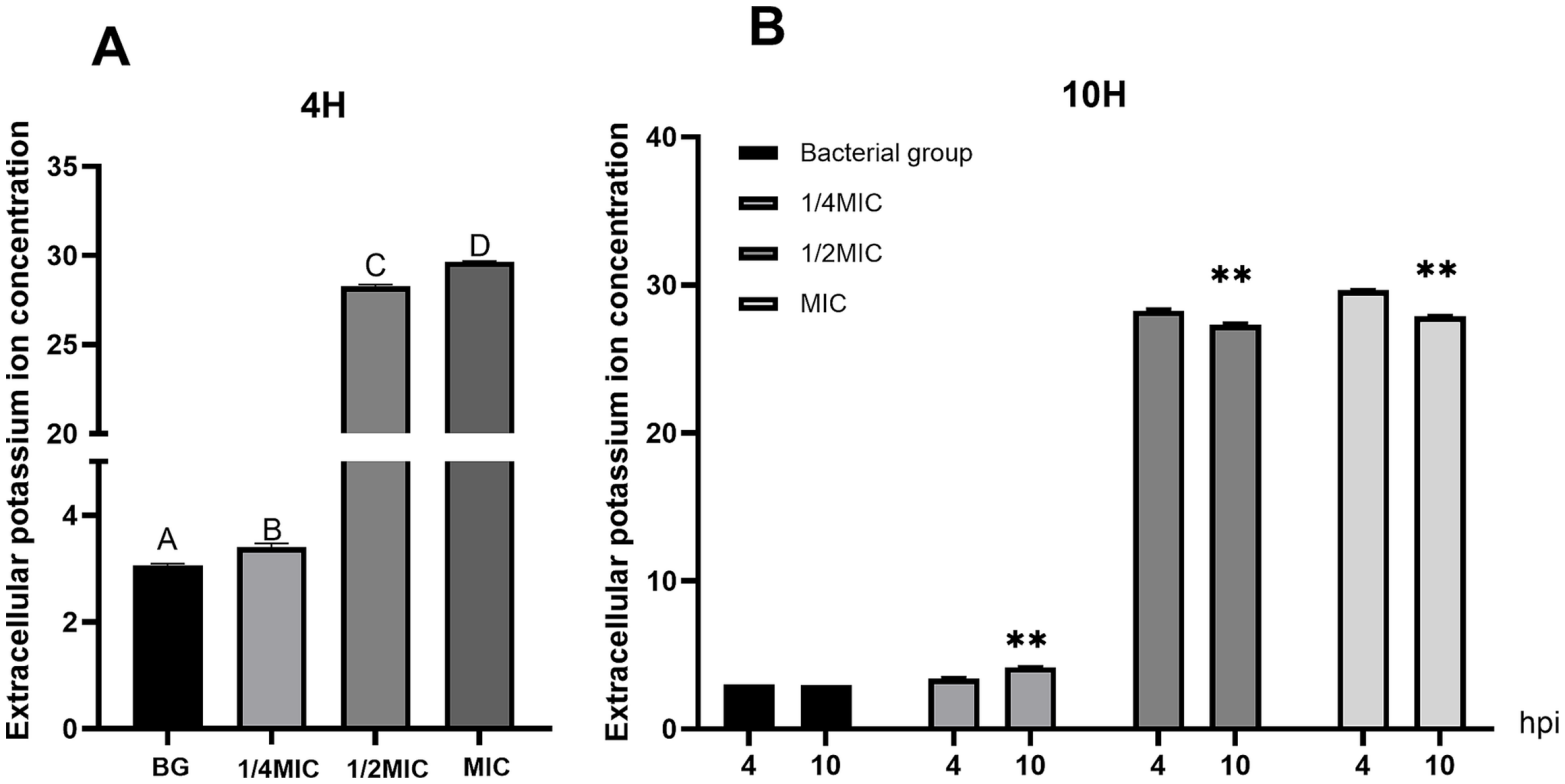
Determination of the extracellular potassium concentration in *Salmonella cholerae*. **(A)** The extracellular potassium concentration at 4h. **(B)** The extracellular potassium concentration at 10h. ** indicates an extremely significant difference (*p*<0.01).

In the untreated bacterial suspension control, the extracellular K^+^ concentrations remained stable at baseline levels, with minimal fluctuations over 4 and 10 h, indicating stable cell membrane integrity. The extracellular potassium ion concentrations in the bacterial culture supernatant were measured at three concentrations (MIC, 1/2 MIC, and 1/4 MIC) at different time points (4 and 10 h). The results revealed that the extracellular K^+^ concentration peaked at 28.280 nmol/L at 4 h post-treatment. Compared with those in the untreated bacterial control (BG) group, the extracellular K^+^ concentrations in the MIC and 1/2 MIC groups increased sharply, whereas those in the 1/4 MIC group were only slightly elevated. At 10 h, statistical analysis of the extracellular K^+^ concentrations revealed extremely significant differences between the MIC group and the other groups (*p* < 0.01) (Figure 4). The K^+^ levels in the MIC and 1/2 MIC groups remained high, and those in the 1/4 MIC group also slightly increased, with all the values being significantly greater than those in the BG group (*p* < 0.01). The MIC group presented the highest potassium ion concentration. These results indicate a clear positive dose- and time-dependent correlation between the extracellular K^+^ concentration and treatment dosage, confirming that the formulation increases potassium ion leakage from *Salmonella* Choleraesuis cell membranes and enhances membrane permeability.

**Figure 5:**
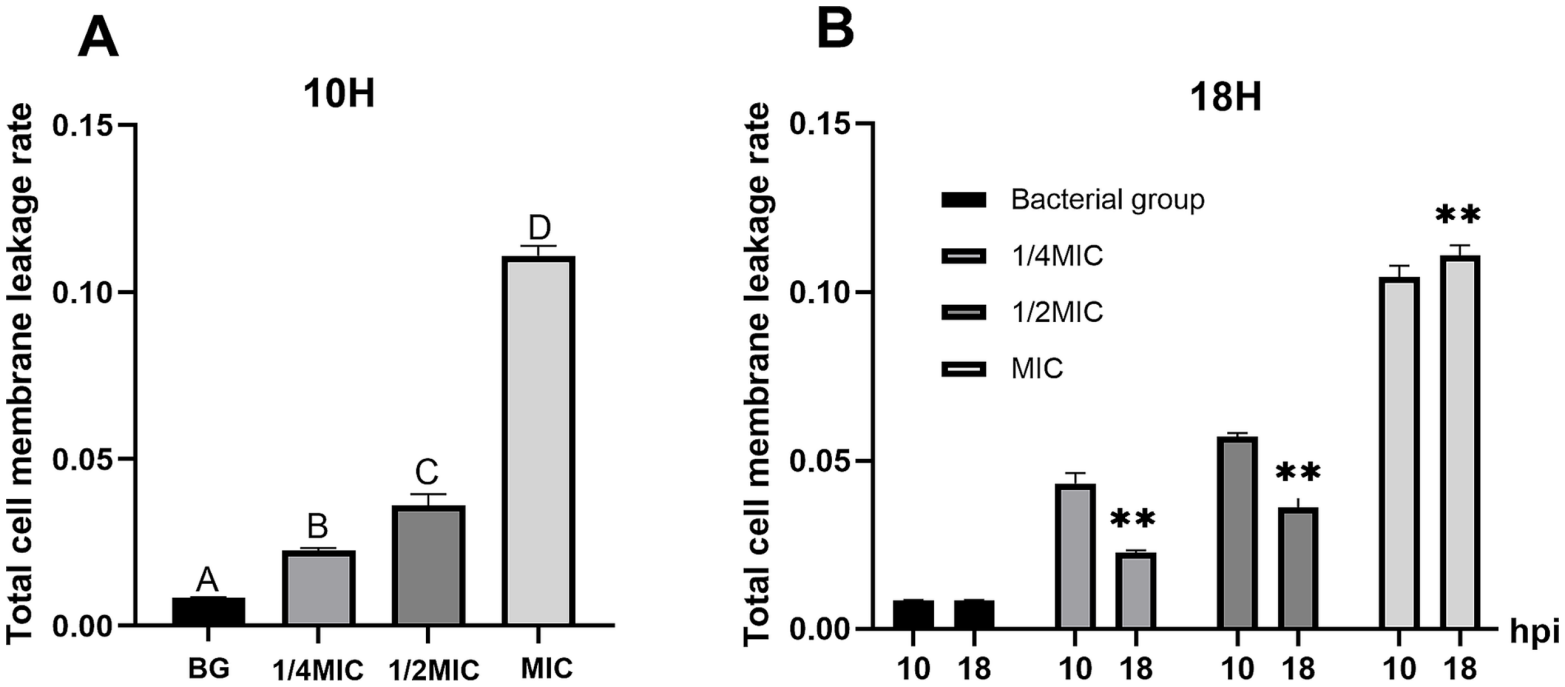
Effects of Chinese herbal compounds on the total leakage rate of *Salmonella choleraesuis*. **(A)** The total leakage rate at 10h. **(B)** The total leakage rate at 18h. The intergroup analysis above was performed, and statistical significance is denoted by **p<0.01 at 18h. At 10h, the intragroup analysis above was performed by letters. The different uppercase letters (A/B/C/D) indicate extremely significant differences (*p*<0.01).

We subsequently evaluated cell membrane permeability by measuring the total cell membrane leakage rate (measured as the OD₆₀₀). In the untreated bacterial control (BG group), the total leakage rate remained stable throughout the 18-h observation period, indicating intact bacterial cell membranes under normal culture conditions. The total leakage rate in the culture supernatant was then quantified at three concentrations (MIC, 1/2 MIC, and 1/4 MIC), and the leakage rate increased with increasing concentration. The OD₆₀₀₀ values in the supernatant of the MIC group continued to rise over the entire 18-h period, whereas those of the 1/2 MIC and 1/4 MIC groups showed a sustained upward trend only within 0–10 h and a downward trend at 18 h. In the intergroup comparison at 10 h, the OD₆₀₀ values of the MIC group were significantly greater than those of the 1/2 MIC and 1/4 MIC groups (*p* < 0.01), and a significant difference was also observed between the 1/2 MIC and 1/4 MIC groups (*p* < 0.01). At 18 h, the leakage rate of the MIC group was the highest, with significant differences compared with those of the other groups (*p* < 0.01) (Figure 5). These findings demonstrate a positive concentration-dependent correlation with the total leakage rate, confirming that the formulation enhances *Salmonella* Choleraesuis cell membrane permeability.

**Figure 6:**
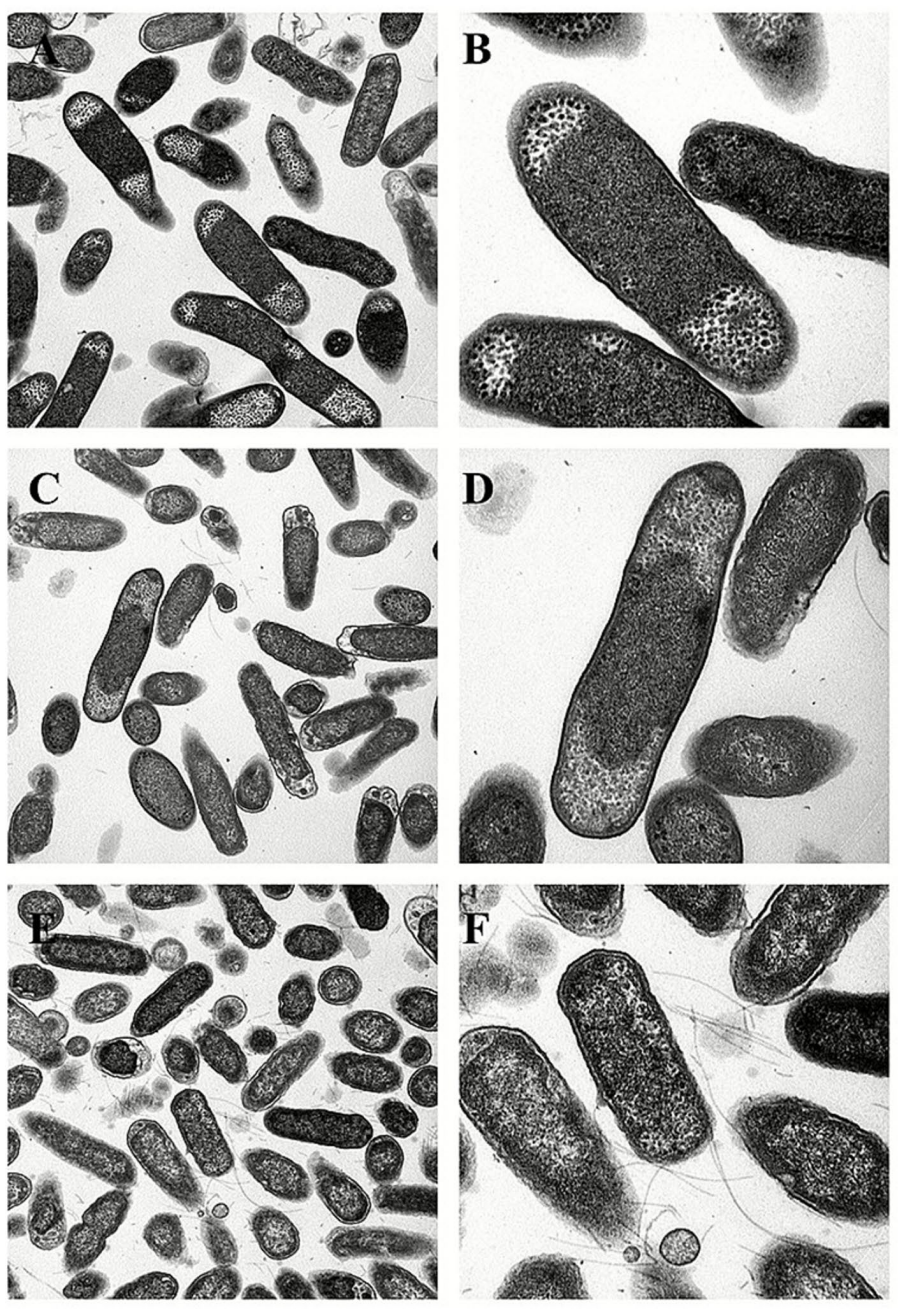
Transmission electron micrographs of the blank control group at 2, 6, and 8 h**. (A,B)** *Salmonella* Choleraesuis blank control group after 2 h (2, 5,000×, 60,000×). **(C,D)** *Salmonella* Choleraesuis blank control group for 6 h (2, 5,000×, 60,000×). **(E,F)** *Salmonella* Choleraesuis blank control group for 8 h (2, 5,000×, 60,000×).

**Figure 7:**
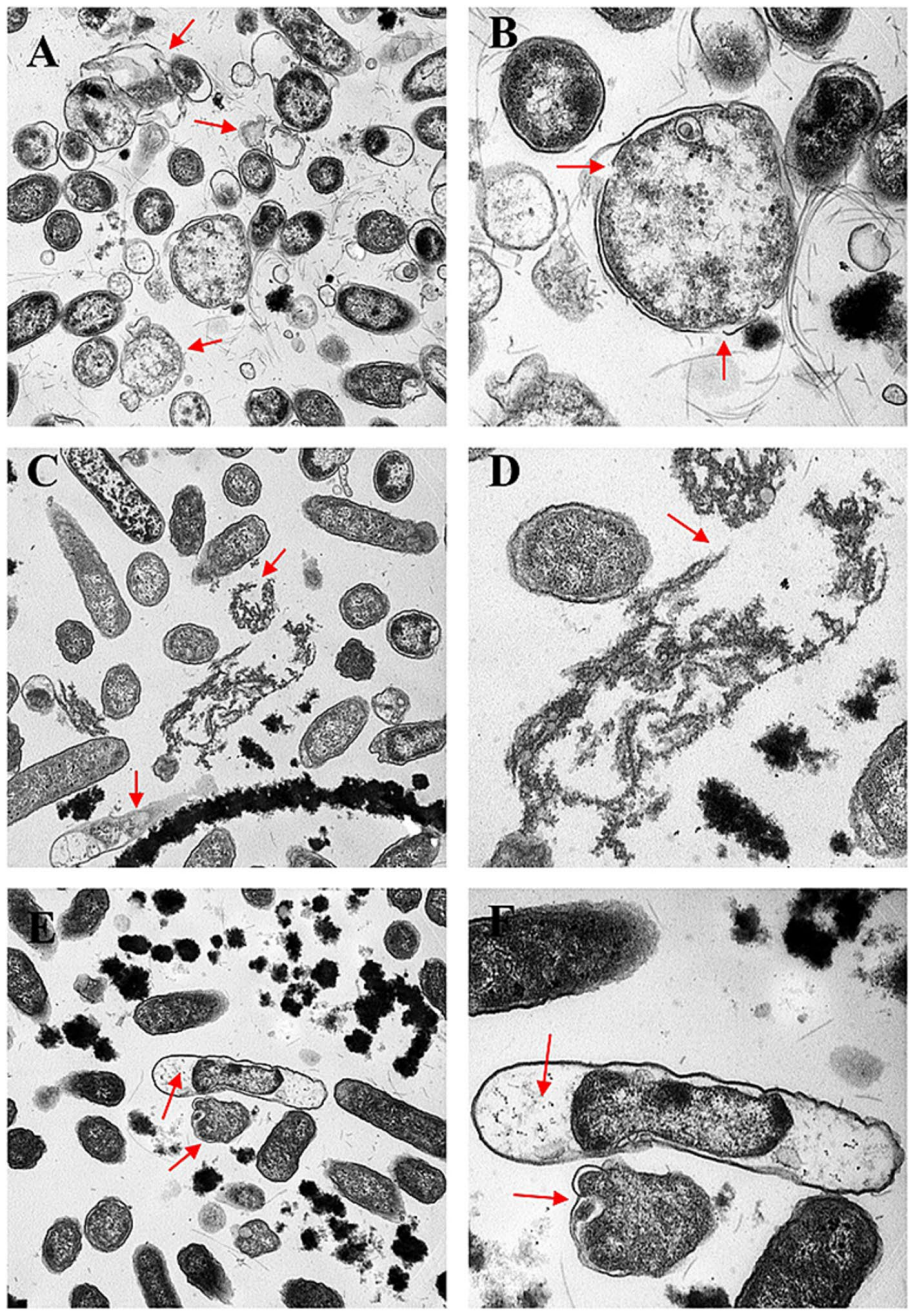
Transmission electron micrographs of the effects of the MICs of Chinese medicinal compounds for 2, 6, and 8 h. **(A,B)** *Salmonella* Choleraesuis MIC traditional Chinese medicine compound group after 2 h (25,000×, 60,000×). **(C,D)** *Salmonella* Choleraesuis MIC traditional Chinese medicine compound group for 6 h (25,000×, 60,000×). **(E,F)** *Salmonella* Choleraesuis MIC traditional Chinese medicine compound group for 8 h (25,000×, 60,000×). The red arrow points to the area damaged by bacteria.

**Figure 8:**
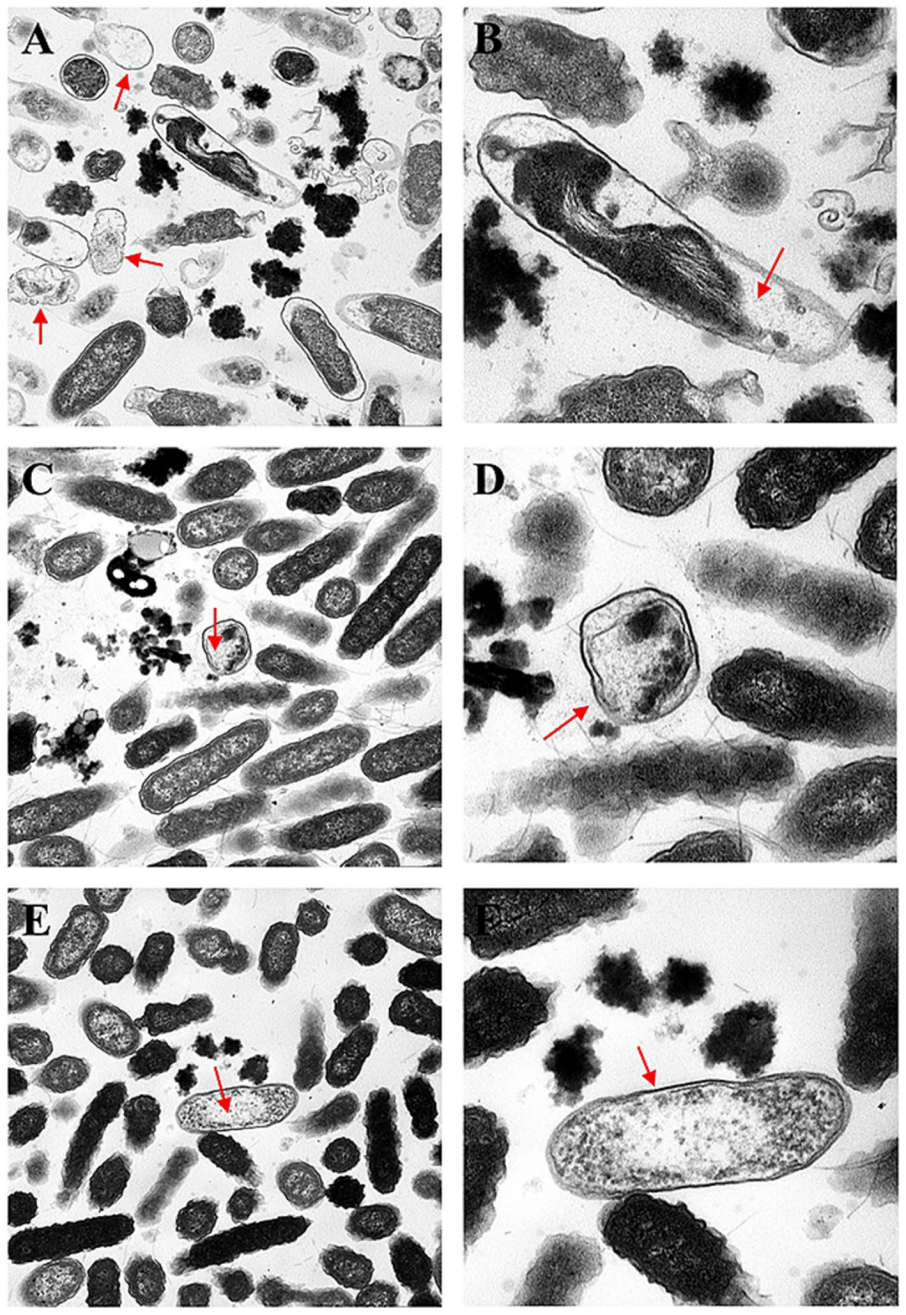
Transmission electron micrographs of the effects of 1/2 MIC Chinese medicine for 2, 6, and 8 h. **(A,B)** *Salmonella* Choleraesuis 1/2MIC traditional Chinese medicine for 2 h (25,000×, 60,000×). **(C,D)** *Salmonella* Choleraesuis 1/2MIC traditional Chinese medicine compound group for 6 h (25,000×, 60,000×). **(E,F)** *Salmonella* Choleraesuis 1/2MIC traditional Chinese medicine compound group for 8 h (25,000×, 60,000×). The red arrow points to the area damaged by bacteria.

**Figure 9:**
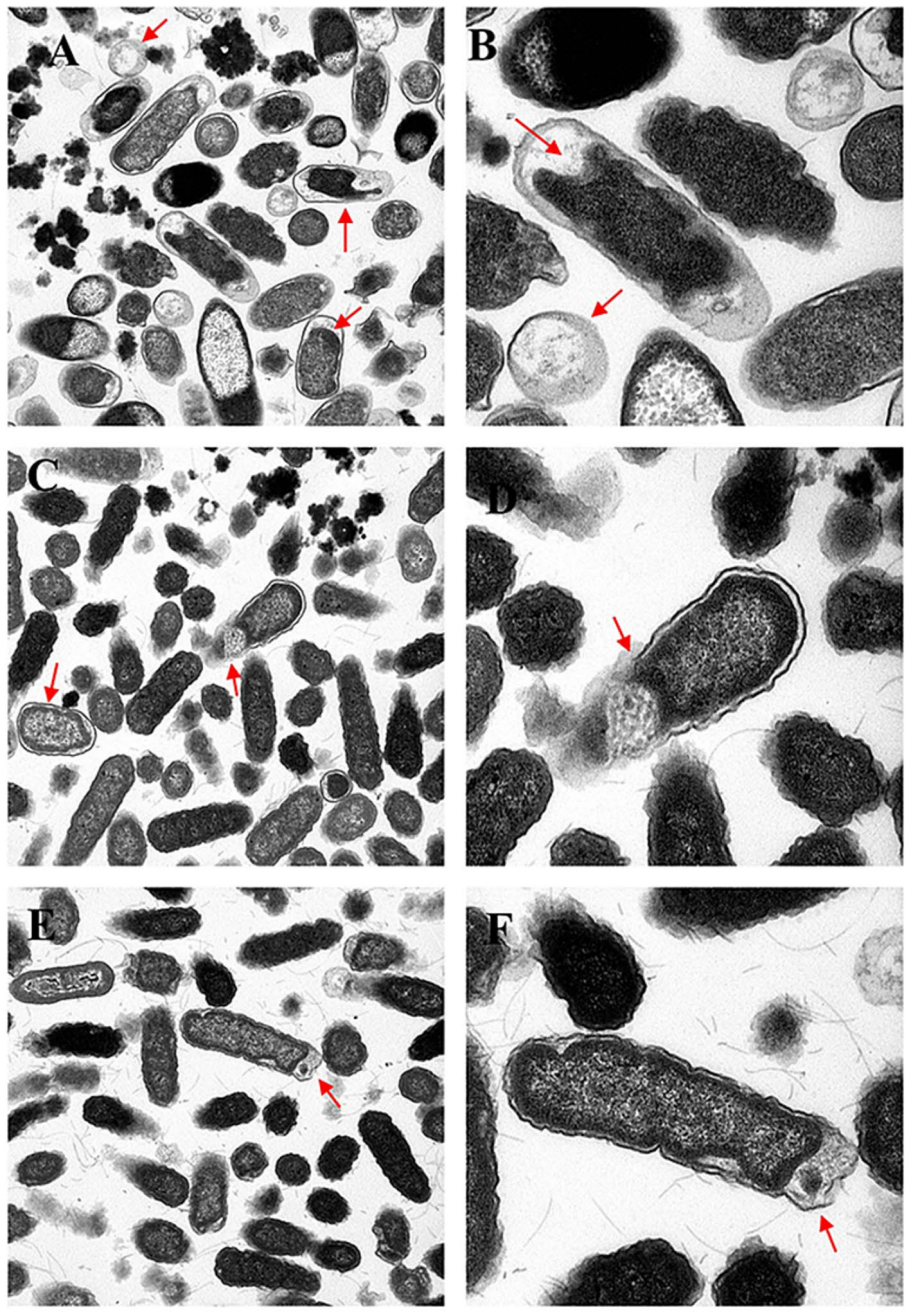
Transmission electron micrographs of Chinese medicine at 1/4 the MIC for 2, 6, and 8 h. **(A,B)** *Salmonella* Choleraesuis 1/4MIC traditional Chinese medicine compound group for 2 h (25,000×, 60,000×). **(C,D)** *Salmonella* Choleraesuis 1/4MIC traditional Chinese medicine compound group for 6 h (25,000×, 60,000×). **(E,F)** *Salmonella* Choleraesuis 1/4MIC traditional Chinese medicine compound group for 8 h (25,000×, 60,000×). The red arrow points to the area damaged by bacteria.

Transmission electron microscopy (TEM) of the untreated control group confirmed that almost 95% of the bacterial cells had an intact ultrastructure, and the preservation of the bacterial ultrastructure was characterized by an intact cell envelope with an apposed cell wall and cytoplasmic membrane, normal flagellar architecture, and uncompromised physiological organization, indicating the maintenance of cellular integrity (Figure 6). In contrast, treatment with three concentrations (MIC, 1/2 MIC, 1/4 MIC) induced progressive and concentration-dependent ultrastructural alterations, as evidenced by TEM analysis at 2, 6, and 8 h post-treatment. In the MIC group, more than 90% of the bacterial cells presented obvious structural damage between the cell wall and the cell membrane, with unevenly distributed cytoplasm, and 85% of the cells formed irregular electron-lucent vacuoles inside the cells (red arrows in Figures 7A,B) at 2 h. The flagella were broken and shed, and the structural integrity of the bacterial cells was damaged. At 6 h, the damage was further aggravated, 40% of the cell walls were dissolved and ruptured, outer membrane fragments were released into the medium, and the cytoplasmic density was significantly decreased (Figures 7C,D). At 8 h, 90% of the bacterial cells had shrunk and deformed, and some even underwent complete lysis (Figures 7E,F). In the 1/2 MIC group, approximately 60% of the bacterial cells presented mild structural damage. The bacterial cells separated from the cell wall and the cell membrane, with small vacuoles present in the cytoplasm and flagella being partially damaged (Figures 8A,B). At 6 h, 40% of the bacteria showed partial recovery from structural damage, accompanied by a reduction in the number of intracellular vacuoles (Figures 8C,D). At 8 h, only a small number of cells displayed uneven cytoplasmic distribution, and the structural damage was repaired (Figures 8E,F). In the 1/4 MIC group at 2 h, 40% of the cells showed slight separation between the cell wall and the cell membrane, with a small number of small vacuoles in the cytoplasm (Figures 9A,B). At 6 h, 40% of the cells exhibited partial recovery from structural damage, with a decreased number of intracellular vacuoles (Figures 9C,D). At 8 h, less than 10% of the bacteria showed very mild structural changes, and 90% of the bacteria tended to have a normal morphology (Figures 9E,F). These results indicate that the severity of bacterial structural damage is positively correlated with both treatment time and drug concentration, suggesting that the formulation has bactericidal activity against *Salmonella* Choleraesuis.

**Figure 10:**
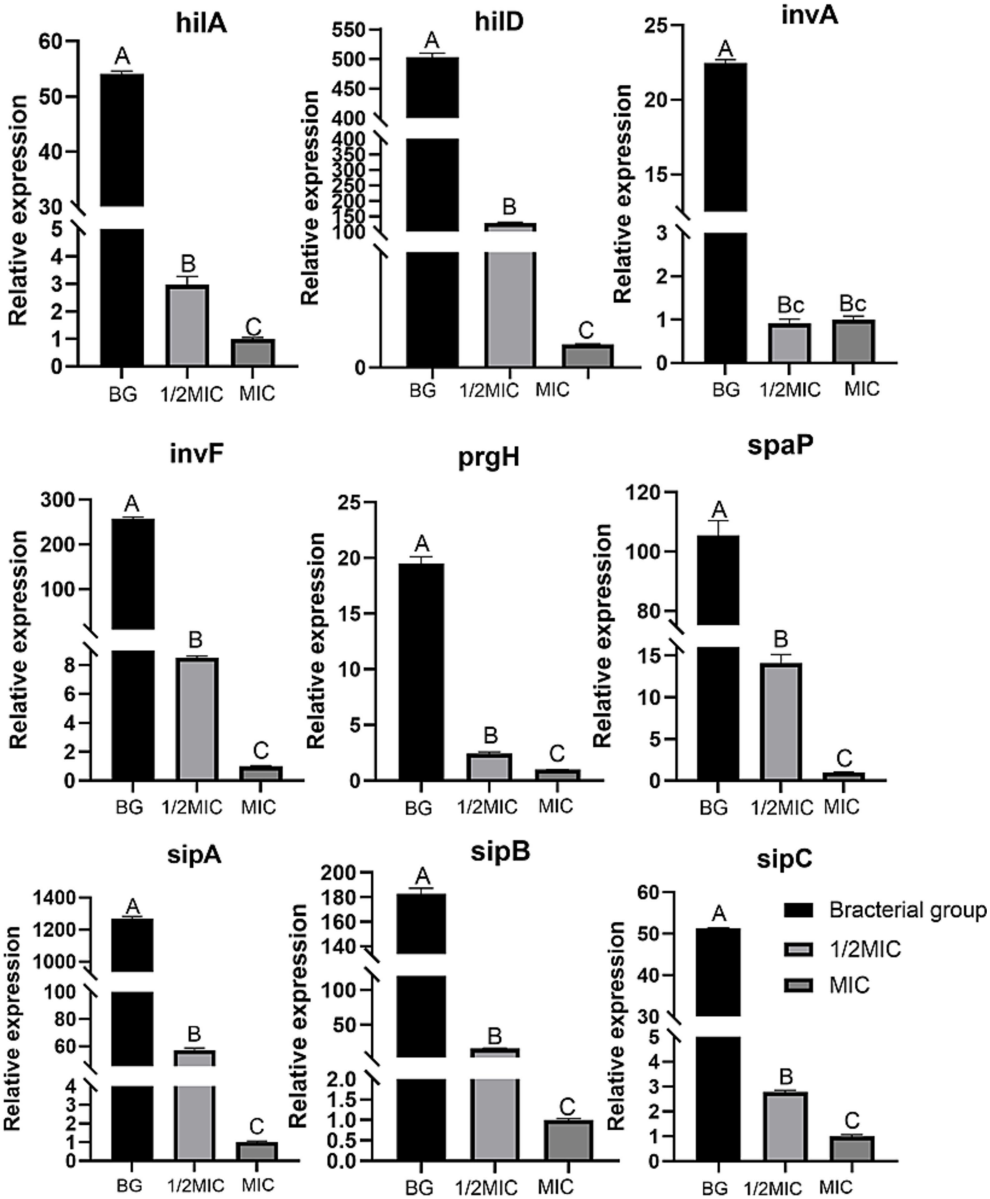
Effect of TCM compounds on the transcription levels of key genes in the bacterial T3SS. The same uppercase letters indicate no significant difference (*p* > 0.05), whereas different uppercase letters indicate extremely significant differences (*p* < 0.01).

The transcriptional levels of key genes in the type III secretion system (T3SS), including sipA, sipB, sipC, invA, invF, prgH, prgI, spaP, hilA, and hilD, were determined at three concentrations (MIC, 1/2MIC, and 1/4MIC) at 10 h. Quantitative real-time PCR (qRT-PCR) analysis revealed that all three concentrations of the formulation significantly downregulated the expression of these genes in *Salmonella* Choleraesuis compared with those in the control group (*p* < 0.01). Notably, the MIC treatment group presented the most pronounced inhibitory effect, followed by the 1/2 MIC group, indicating a clear dose-dependent response (Figure 10). The transcriptional levels of key genes in the type III secretion system (T3SS), including sipA, sipB, sipC, invA, invF, prgH, prgI, spaP, hilA, and hilD, were determined at three concentrations (MIC, 1/2MIC, and 1/4MIC) at 10 h. Quantitative real-time PCR (qRT-PCR) analysis revealed that all three concentrations of the formulation significantly downregulated the expression of these genes in *Salmonella* Choleraesuis compared with those in the control group (*p* < 0.01). Notably, the MIC treatment had the most pronounced inhibitory effect, followed by the 1/2 MIC treatment, indicating a clear dose-dependent response (Figure 10). Among the upstream regulatory genes of the T3SS, hilA and hilD presented the most significant downregulation in the MIC group. The downregulation of these two genes blocks the activation of the entire T3SS virulence gene network, which is the core of the formula’s ability to inhibit bacterial virulence. The transcription levels of sipA, sipB and sipC (the core effector proteins of the T3SS mediating bacterial invasion) in the MIC group were decreased. The transcription levels of prgH (needle complex structural gene) and spaP (secretion apparatus gene) in the MIC group decreased, which led to the loss of T3SS structural integrity and the inability to secrete effector proteins. The inhibitory effect was ranked as the MIC > 1/2 MIC. This consistent dose–response relationship across all key T3SS genes strongly indicates that the formulation effectively and concentration-dependently suppresses the transcriptional activation of the T3SS in *Salmonella* Choleraesuis.

### Establishment of a mouse diarrhea model

**Figure 11:**
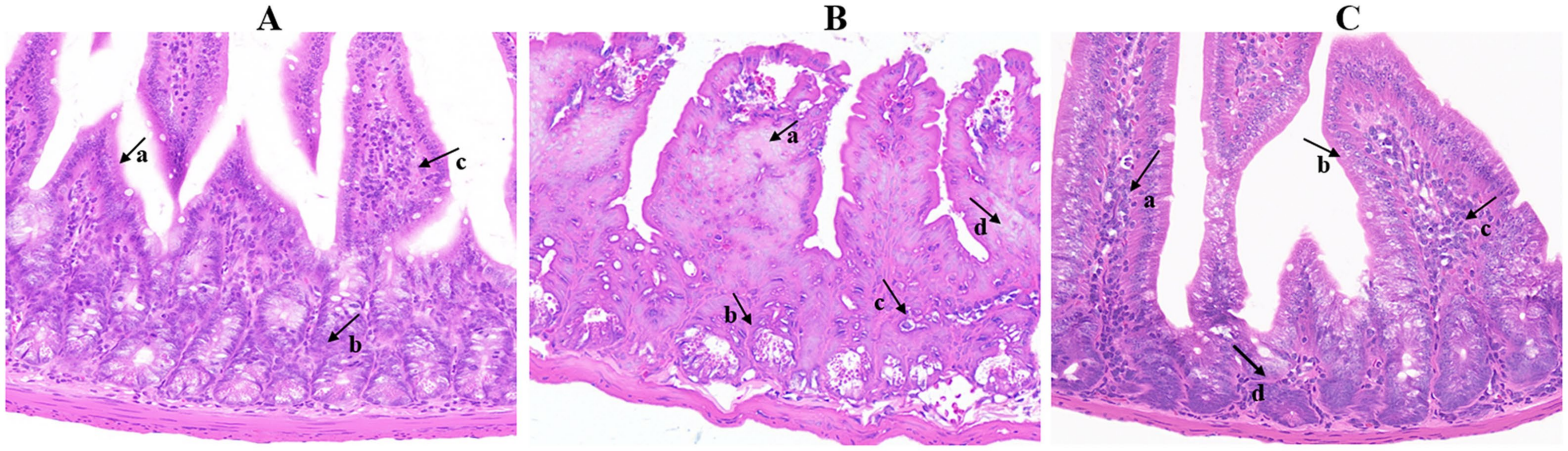
Histopathological analysis of mouse intestines (20×). **(A)** Control group (the villi and crypts were normal), **(B)** bacterial group (the villus structure in the infection group was disordered and fractured; the crypts were damaged with irregular structures). **(C)** TCM group: the visceral lesions were milder, with reduced intestinal gland damage and alleviated intestinal congestion.

The LD_50_ of *Salmonella* Choleraesuis was 9.9 × 10^4^ CFU/mL. Animal regression tests revealed that mice infected with *Salmonella* Choleraesuis developed typical diarrhea symptoms, with 100% mortality observed within 7 days post-infection. Pathological dissection revealed multiorgan lesions, and the pathogenic bacteria were confirmed by isolation and identification. Challenge tests with different concentrations revealed that a bacterial suspension of 5 × 10^4^ CFU/mL induced consistent diarrhea symptoms in mice with minimal mortality; thus, this concentration was determined to be the optimal challenge concentration (Figure 11).

### Efficacy evaluation of the Chinese herbal formulation in a mouse diarrhea model

In this study, a mouse diarrhea model was established using *Salmonella* Choleraesuis, and the experimental mice were randomly divided into four groups: the control group, the bacterial infection group, the Chinese herbal compound group (10 g/kg), and the antibiotic group (10 g/kg). The mice in all the bacterial infection groups were subjected to intragastric administration for 7 consecutive days. The therapeutic efficacy of the Chinese herbal compound was preliminarily evaluated by detecting the mouse survival rate, routine blood parameters, secretory immunoglobulin A (sIgA) levels, and the mRNA expression levels of key proinflammatory cytokine genes (IL-1β, IL-6, and TNF-α).

**Figure 12:**
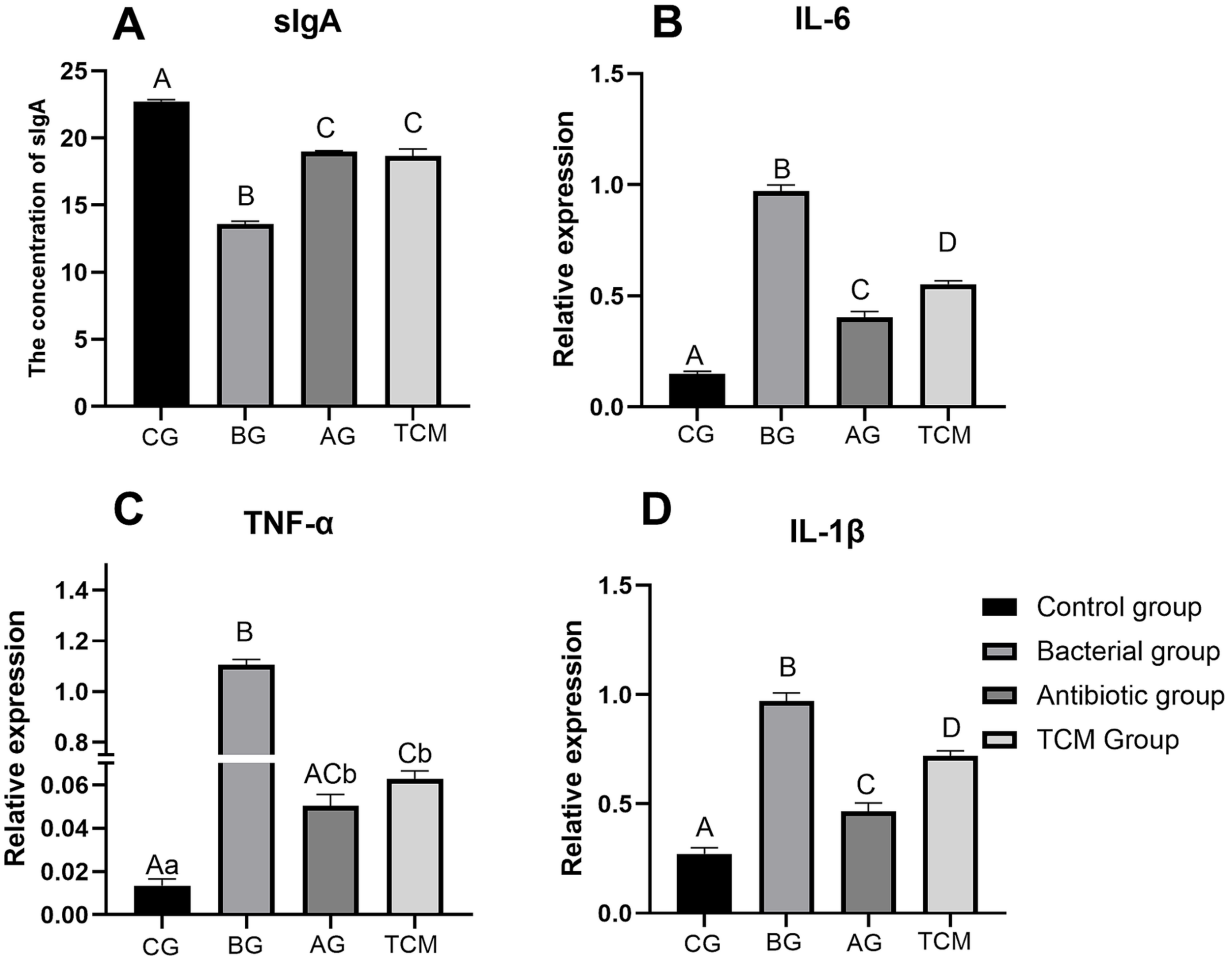
Effects of traditional Chinese medicine compounds on the sIgA and inflammatory factors of the mice. Different uppercase letters (A/B/C/D) indicate extremely significant differences among groups (*p*<0.01); different lowercase letters (a/b) indicate significant differences among groups (0.05<*p*<0.01).

The experimental results revealed that both the antibiotic group (10 g/kg) and the Chinese herbal compound group (10 g/kg) presented relatively high mouse protection rates, with the protection rate of the Chinese herbal compound group reaching 66.7%. In terms of sIgA levels, the concentration in the Chinese herbal compound group was significantly greater than that in the bacterial model group (*p* < 0.01) (Figure 12A). In addition, the mRNA expression levels of IL-6 (Figure 12B), TNF-α (Figure 12C), and IL-1β (Figure 12D) in the blood of the mice in the three treatment groups were significantly lower than those in the bacterial infection model group (*p* < 0.01).

Histopathological observations revealed that the villus structure in the infection group was disordered and fractured; the crypts were damaged with irregular structures, and some crypt epithelial cells were degenerated. In contrast, the degree of visceral lesions in the mice in the treatment groups was milder than that in the infection model group, with reduced intestinal gland damage, relieved intestinal congestion, and significantly improved intestinal adhesion.

In conclusion, these findings confirm that this Chinese herbal compound has a therapeutic effect on diarrhea induced by *Salmonella* Choleraesuis in mice.

## Discussion

### Innovation and advantages of screening single TCM herbs and optimizing formulations against *Salmonella* Choleraesuis

Against the backdrop of the comprehensive ban on antimicrobial growth promoters in feed, the livestock industry’s demand for “antibiotic alternatives” is increasing. The development of feed additives with both disease-resistant and growth-promoting properties has emerged as a core research focus in the field of “antibiotic substitution.” On the basis of the Chinese Catalog of Feed Raw Materials, this study selected 10 types of medicinal and edible homologous TCMs for *in vitro* antibacterial assays.

The results revealed that *Mume Fructus*, *Schisandrae Chinensis Fructus*, and *Magnoliae officinalis* exhibited significant antibacterial effects. Among these, *Schisandrae Chinensis Fructus* had the strongest antibacterial activity, followed by *Mume Fructus* and *Magnoliae officinalis*. *Crataegi Fructus*, *Atractylodes lancea*, and *Lophatheri Herba* had relatively weak antibacterial effects, whereas *Dolichos lablab*, *Lonicerae japonica Flos*, *Agastaches Herba*, and *Psoraleae Fructus* had no significant antibacterial activity.

In pairwise combination tests, 5 groups of combinations, such as *Mume Fructus-magnolia officinalis* (FIC = 0.625) and *Crataegi Fructus-Atractylodes rhizome* (FIC = 0.625), had synergistic effects, 10 groups had additive effects, and there were no antagonistic combinations. This finding indicates that the compound prescriptions composed of the selected single medicines have no antagonistic effect, laying a foundation for subsequent prescription screening and avoiding the limitation of the narrow antibacterial spectrum of single drugs.

Orthogonal testing has become a core technology for traditional Chinese medicine (TCM) compound screening because of its relatively few experimental runs and high accuracy of results. In this study, the results revealed that *Schisandra chinensis*, *Mume Fructus*, *Atractylodes lancea*, and *Crataegus pinnatifida* played key antibacterial roles. Further optimization of the dosage was performed via the L9 (3^4^) orthogonal test, and the optimal compounds were determined as follows: *Mume Fructus,* 30 g; S*chisandra chinensis,* 10 g; *Crataegus pinnatifida,* 5 g; and *Atractylodes lancea,* 5 g. Benefiting from the high accuracy of dosage optimization, the dosage combination (*Mume Fructus,* 30 g; *Schisandra chinensis,* 10 g) identified by this orthogonal test not only ensures antibacterial efficacy but also significantly reduces the dosage of the high-dose medicinal material (*Atractylodes lancea*). Additionally, the optimal formulation exhibited excellent antibacterial activity, with a minimum inhibitory concentration (MIC) of 15.625 mg/mL and a minimum bactericidal concentration (MBC) of 31.25 mg/mL.

### Antibacterial mechanisms of traditional Chinese medicine compounds against *Salmonella* Choleraesuis

The selective permeability of bacterial cell membranes and the integrity of the cell structure are crucial for maintaining bacterial survival and metabolic homeostasis. Once damaged, it leads to the leakage of intracellular substances and the disruption of metabolic processes, thereby exerting antibacterial effects. This study explored the antibacterial mechanism of traditional Chinese medicine compounds by monitoring their extracellular potassium ion concentrations and OD600 values and observing their bacterial ultrastructures.

In the extracellular K^+^ leakage assay, the extracellular K^+^ concentrations in the MIC (15.625 mg/mL), 1/2MIC (7.813 mg/mL), and 1/4MIC (3.906 mg/mL) groups peaked at 29.656 nmol/L, 28.280 nmol/L, and 14.151 nmol/L, respectively, after 4 h of drug exposure, with significant intergroup differences (*p* < 0.01). These data demonstrate a positive correlation between extracellular K^+^ levels and the concentrations of the TCM formulation. The OD_600_ values revealed that the MIC group continued to increase over 18 h, whereas the 1/2MIC and 1/4MIC groups decreased during the same period, confirming that the total leakage rate of the bacterial suspension was correlated with the concentration of the TCM formulation and further verifying that the formulation enhances the permeability of *Salmonella* Choleraesuis cell membranes.

The cell wall and cell membrane of *Salmonella* species serve as pivotal barriers for maintaining structural and functional integrity. Transmission electron microscopy (TEM) revealed concentration-dependent and time-dependent ultrastructural damage. In the MIC group, most bacterial cells exhibited detachment between the cell wall and the cell membrane, with irregular cytoplasmic vacuolization at 2 h. At 6 h, localized dissolution of the cell wall was observed, accompanied by the release of outer membrane fragments. At 8 h, the cell membrane displayed irregular folding, and the cytoplasmic electron density became heterogeneous. In the 1/2 MIC group, mild cytoplasmic vacuolization was noted at 2 h, with partial structural recovery by 6 h. The 1/4 MIC group presented the minimal extent of damage.

These observations are consistent with the findings of Hong et al., who reported structural damage to *Escherichia coli* and Salmonella following treatment with *Rheum palmatum* L., Scutellaria baicalensis Georgi, and *Houttuynia cordata* Thunb (Jiang et al., 2023). Collectively, these results indicate a positive correlation between the degree of bacterial structural damage and both the drug concentration and exposure duration. This discovery revises the conventional perception of TCMs as “slow-acting bacteriostatic agents” and validates their ability to rapidly permeate bacterial barriers, with a kinetic profile comparable to that of antibiotics (e.g., enrofloxacin can induce bacterial damage within 2.4 h of exposure) (Wu et al., 2006). Bacterial structural damage leads to disruption of the cell wall peptidoglycan layer, the release of outer membrane fragments, and the formation of cytoplasmic vacuoles, which further cause the collapse of the transmembrane potential. This study also elucidates the core mechanism underlying the “multitarget synergistic disruption” of bacterial cellular structures by TCM compounds.

### Effect of the TCM formulation on the transcription of key T3SS genes in *Salmonella* Choleraesuis

The pathogenicity of *Salmonella* is highly dependent on the type III secretion system (T3SS), which is encoded by SPI-1 and SPI-2 and mediates processes such as adhesion and invasion through the secretion of effector proteins. This study did not use SPI-2-encoded T3SS genes because the SPI-2 T3SS is mainly responsible for the survival and proliferation of *Salmonella* in macrophages and systemic infection, whereas the main infection form of *Salmonella* Choleraesuis in the early stage of piglet infection is intestinal mucosal infection (mediated by the SPI-1 T3SS). This study focused on the antibacterial effect of the compound prescription on the intestinal infection stage of *Salmonella* Choleraesuis, so the SPI-1 T3SS core genes are key research objects. In the present study, the TCM formulation at the MIC, 1/2 MIC, and 1/4 MIC significantly downregulated the transcriptional levels of key T3SS genes in *Salmonella* Choleraesuis (*p* < 0.01), with the strongest inhibitory effect observed in the MIC group. hilA and hilD are regulatory genes; prgH, spaP, invA, and invF are structural genes; and sipA, sipB, and sipC are effector protein genes.

At the level of effector protein genes, the transcription of *sipA* was initially downregulated after 2 h of exposure to the MIC, with expression being suppressed to undetectable levels by 10 h, leading to reduced synthesis of actin-binding proteins and marked impairment of membrane ruffle formation capacity. The transcription of *sipB* and *sipC* was downregulated at 4 h, disrupting needle complex assembly. The expression of the invasion genes *invA* and *invF* was downregulated after 2 h, inhibiting bacterial penetration. The expression of the apparatus genes *prgH* and *spaP* decreased after 2 h, leading to the loss of T3SS structural integrity. The transcription of the regulatory genes *hilA* and *hilD* was significantly downregulated at 2 and 4 h, respectively, blocking the virulence gene activation network at the upstream level. These results indicate that the TCM formulation can simultaneously block the SPI-1 (invasion)-encoded type III secretion system (T3SS), enabling intervention across the entire infection process, from the initial stage (adhesion to M cells) to systemic dissemination. Compared with more extensively studied single-target agents (SPI-1-specific inhibitors such as cinnamaldehyde) (Liu et al., 2019; Veenendaal et al., 2009; Tsou et al., 2013), the multitarget inhibition of T3SS gene expression results in superior efficacy.

The formulation disrupts the pathogenic machinery of *Salmonella* Choleraesuis at multiple levels, including effector protein secretion, activation of invasion-associated genes, functionality of regulatory networks, and structural assembly.

### Effects of the TCM formulation on intestinal secretory immunoglobulin A and the transcription levels of inflammatory cytokines in diarrheal mice

All the herbs in the optimal formula (*Mume Fructus*, *Schisandrae Chinensis Fructus*, *Crataegi Fructus*, *Atractylodes lancea*) are listed in the Catalog of Feed Raw Materials issued by the Ministry of Agriculture and Rural Affairs of China, with clear food and feed safety attributes. In our preliminary acute toxicity test, the maximum tolerated dose (MTD) of the compound prescription extract in mice was more than 20 g/kg (crude drug dose), which is twice the therapeutic dose used in the study, indicating good *in vivo* safety. TCM compounds possess the advantages of multicomponent and multitarget synergistic actions in veterinary clinical antimicrobial therapy. The results revealed that the 10 g/kg TCM group achieved a 66.7% protection rate in mice. Histopathological observations revealed that the villus structure of the infection group was disordered and fractured, the crypts were damaged and structurally irregular, and some crypt epithelial cells were degenerated. In contrast, the intestinal lesions of the treatment group were significantly milder, with reduced intestinal gland damage, alleviated intestinal congestion, and significantly improved intestinal adhesion.

According to the body surface area conversion method for animal dose conversion, the crude drug dose of 10 g/kg in mice is converted to approximately 0.8–1.0 g/kg in pigs, which can be further adjusted to 0.5–1.0% of the feed mass (i.e., 5–10 g/kg feed) in actual pig breeding. This addition ratio is within the common range of Chinese herbal feed additives in the livestock industry and has good economic feasibility. Additionally, the intestinal sIgA concentrations in the TCM group were significantly greater than those in the bacterial model (BG) group (*p* < 0.01). The underlying mechanism may be related to the promotion of the differentiation of B cells into IgA plasma cells in intestinal Peyer’s patches and the upregulation of the expression of polymeric immunoglobulin receptors (Fagarasan et al., 2010; Cyster and Allen, 2019; Bunker and Bendelac, 2018; Santos et al., 2001; Bunker and Bendelac, 2018). These results indicate that traditional Chinese medicine compounds can not only directly kill bacteria and block their damage to the intestinal mucosa but also activate the intestinal mucosal immune response in mice, enhance the local immune barrier function of the intestine, and thus promote the repair of intestinal mucosal damage. This finding is consistent with the multicomponent characteristics of the compound. Network pharmacology analysis revealed that *Mume Fructus*, *Schisandra chinensis*, *Atractylodes lancea*, and *Crataegi pinnatifida* contain multiple anti-inflammatory active components, among which quercetin has the highest degree value, and these components may synergistically play a role in protecting the intestinal mucosa.

*Salmonella* infection can trigger a cascade of inflammatory factors in the body. This study revealed that, after treatment with traditional Chinese medicine, the gene expression levels of interleukin-6 (IL-6), tumor necrosis factor-α (TNF-α), and interleukin-1β (IL-1β) in the compound group and the antibiotic group were significantly lower than those in the bacterial infection model group (*p* < 0.01). Combined with the network pharmacology and molecular docking results, this anti-inflammatory effect may be closely related to the regulation of key inflammatory targets and signaling pathways by the active components of the compound. Hubbs network analysis identified 91 core inflammatory targets, among which TNF and IL-6 are key targets. Core targets refer to the protein targets that are directly bound by the active components of the Mume Fructus-Schisandra formula and are significantly involved in the inflammatory response and anti-*S. choleraesuis* infection process of the host. The targets were obtained by the intersection of the potential targets of the active components of the formula (retrieved from the SRPLOT platform and RCSB PDB). After the protein–protein interaction network was constructed, the number of targets with a degree value > the median value of all intersection targets was determined. KEGG pathway enrichment analysis revealed that these targets are associated mainly with the TNF signaling pathway, which is involved in the inflammatory response. Molecular docking verification further confirmed that quercetin and isorhamnetin (derived from *Mume Fructus* and *Crataegi Fructus*) have high affinities for IL-6 and TNF-α: IL-6 (PDB ID: 1IL6) has the same high affinity for quercetin and isorhamnetin, with a binding energy of −7.2 kcal/mol; TNF-α (PDB ID: 1TNF) has the highest affinity for isorhamnetin, with a binding energy of −10.8 kcal/mol. The stable binding of these components to targets is mediated by hydrogen bonds and hydrophobic bonds. Consistent with the experimental results, the traditional Chinese medicine compounds reduced the transcriptional levels of these inflammatory factors. The cascade of inflammatory factors induced by *Salmonella* infection plays an important role in the pathogenic process. This study revealed that the gene expression levels of IL-6, TNF-α, and IL-1β in the TCM group and antibiotic group (AG) were significantly lower than those in the model group (*p* < 0.01). Among them, the downregulated expression of IL-6 suggests that TCM compounds can inhibit the activation of the NF-κB signaling pathway, whereas the dose-dependent downregulation of TNF-α and IL-1β is related to the regulation of the TLR4/MyD88 pathway, which aims to inhibit the excessive inflammatory response induced by bacterial infection and alleviate inflammatory damage to intestinal and systemic tissues.

In summary, the network pharmacology and molecular docking analyses in this study were designed to explore the molecular mechanism by which the compound prescription regulates the host immune response (anti-inflammatory effect), and the direct antibacterial mechanisms (membrane damage and virulence inhibition) were verified via *in vitro* bacterial cell experiments (including extracellular potassium ion concentration detection, total leakage rate measurement, transmission electron microscopy (TEM) observation, and RT-qPCR detection of T3SS gene transcription levels). These are two independent research parts that explain the direct antibacterial effect and host immune regulatory effect of the compound prescription, and together constitute the multitarget antibacterial mechanism of the formula. This TCM formulation exhibits dual efficacy, combining “bacteriostasis+immunoregulation,” with a strength comparable to that of veterinary antibiotics (e.g., enrofloxacin). Moreover, it avoids issues of antibiotic residues and drug resistance. From the perspective of industrial application, the formulation can achieve good anti-inflammatory effects at a dose of 10 g/kg, reducing costs, and its preparation process is simple, making it suitable for large-scale production.

NP532 was administered as an intervention to chicks after challenge with Salmonella gallinarum. NP532 is a methanol extract of a Chinese herbal formula consisting of Mume Fructus, Coptidis Rhizoma, and Schisandrae Fructus for the treatment of salmonellosis. The results revealed that the NP532 significantly reduced the mortality of infected chickens (Kwon et al., 2008). Although NP532 exhibited favorable antibacterial efficacy, the preparation process—including extraction of NP532 from the formula and subsequent formulation into feed additives—was time-consuming and costly. Enrofloxacin, an antibiotic, is widely used in clinical treatment; however, its long-term application can induce the emergence of multidrug-resistant *Salmonella* Choleraesuis strains. Moreover, enrofloxacin has a long half-life in animal tissues and tends to cause drug residues. Compared with the preventive and therapeutic effects of single compounds and antibiotics, the present formula has greater advantages. As a multicomponent formulation, it exerts its effects through multiple targets, including cell membrane damage, virulence inhibition, and immunoregulation, making it difficult for bacteria to develop drug resistance. In contrast, enrofloxacin only possesses antibacterial activity without any immunoregulatory effects and may even exert certain inhibitory effects on the host immune system at high doses. The present formula not only kills bacteria but also increases intestinal sIgA levels, downregulates the expression of proinflammatory cytokines, enhances the host’s intestinal mucosal immunity, and reduces the secondary infection rate in diseased animals.

In addition, the selected medicinal materials, such as *Mume Fructus*, *Schisandra chinensis*, and *Crataegi pinnatifida,* are included in the Chinese Catalog of Feed Raw Materials, with clear safety and a legal basis for feed application. In combination with the *in vitro* antibacterial activity, *in vivo* therapeutic effect, and clear mechanism of action confirmed in this study, this traditional Chinese medicine provides important data support and a practical basis for the “antibiotic substitution” scheme in pig breeding. It is expected to be developed into a new feed additive for the prevention and control of *Salmonella* Choleraesuis infection, promoting the healthy and sustainable development of the livestock industry.

## Conclusion

Through *in vitro* bacteriostatic tests and orthogonal test screening and optimization, a four-herb Chinese medicine mixture consisting of 30 g of *Fructus Mume*, 10 g of *Schisandra chinensis*, 5 g of *Crataegi Fructus* and 5 g of *Atractylodes lancea* was obtained. This Chinese medicinal compound has bacteriostatic effects on *Salmonella* Choleraesuis. It acts by altering the permeability of bacterial cell membranes, damaging the bacterial cell structure, and inhibiting the transcription of key genes in the type III secretion system (T3SS). The compound demonstrated effective therapeutic effects in mice with diarrhea caused by *Salmonella* Choleraesuis.

## Data Availability

The original contributions presented in the study are included in the article/Supplementary material, further inquiries can be directed to the corresponding authors.

## Footnotes

1
https://www.rcsb.org/
