# Supplementary material for: Preventing Salmonella Choleraesuis infection by Mume Fructus-Schisandra formulas through the “membrane damage-virulence inhibition-immunomodulation” pathway
Source: Front Microbiol. 2026 Apr 14;17:1781674. doi: 10.3389/fmicb.2026.1781674 (PMC13121317; doi:10.3389/fmicb.2026.1781674)
Supplement: Supplementary file 1 [file Data_Sheet_1.DOCX]

**Supplementary figures**


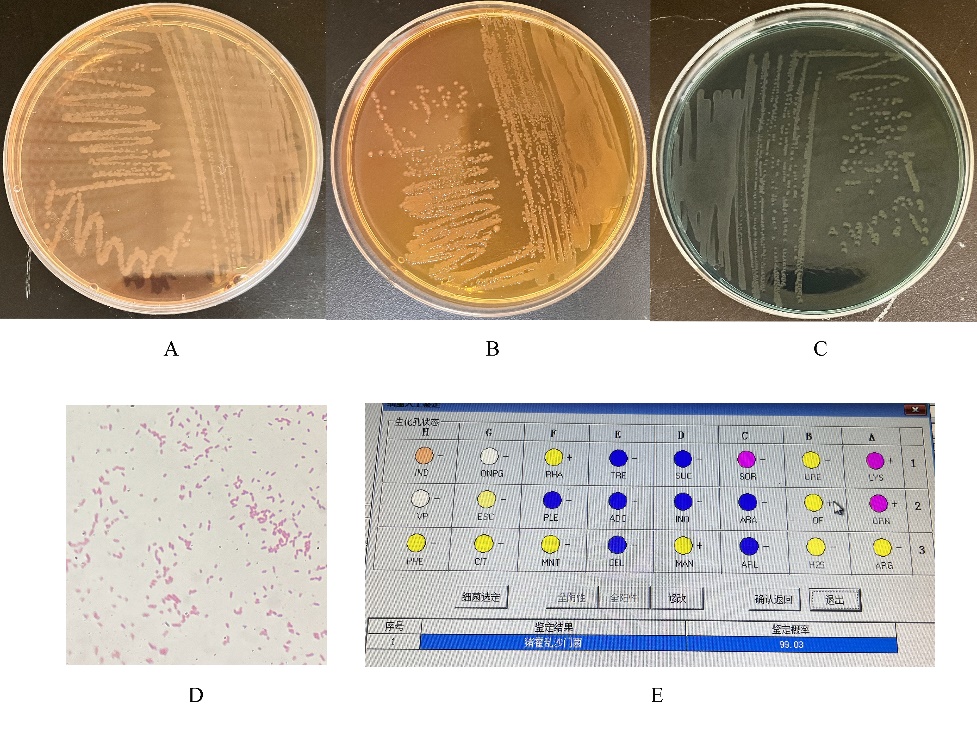


**Supplementary Figure 1. Isolation and identification of Salmonella cholerae in pigs.**

**Note:** A: Isolation and culture on MacConkey agar media; B: Isolation and culture on SS agar media; C: Isolation and culture on WS agar media; D: Gram staining and microscopic examination; E: Identification results via bacterial identification and a drug sensitivity analyzer

**Supplementary tables**

**Table 1.** Diarrhea rates and mortality in mice.

| **Group** | **Dosage** | **Number of animals（pieces）** | **Number of deaths (pieces)** | **Number of survivors (pieces)** | **Mortality rate (%)** | **Protection rate (%)** |
| --- | --- | --- | --- | --- | --- | --- |
| Control group | 0 | 10 | 0 | 10 | 0 | - |
| Model group | 0 | 10 | 3 | 7 | 30 | - |
| Antibiotic group | 10g/kg | 10 | 0 | 10 | 0 | 100 |
| TCM group | 10g/kg | 10 | 1 | 9 | 10 | 66.7 |

**Table 2.** Routine blood indices of the mice after 7 days of treatment.

| Group | WBC（10^9^/L） | LYM(%) | NEU% | MON(%) | RBC（10^12^/L） | HGB(g/L) |
| --- | --- | --- | --- | --- | --- | --- |
| Control group | 3.47±0.38^b^ | 76.29±2.47^a^ | 22.36±3.01^d^ | 3.74±0.44^d^ | 8.49±0.36^a^ | 153.71±8.65^a^ |
| Bacterial group | 4.27±0.29^a^ | 21.37±1.26^d^ | 41.73±1.87^a^ | 23.06±1.10^a^ | 7.24±0.16^b^ | 126.43±2.70^b^ |
| Antibiotic group | 3.48±0.36^b^ | 37.81±2.78^b^ | 36.73±1.89^b^ | 15.87±1.02^c^ | 6.15±0.36^c^ | 110.00±5.77^c^ |
| TCM group | 3.33±0.79^b^ | 34.61±2.57^c^ | 27.43±3.05^c^ | 17.73±1.06^b^ | 7.20±0.49^b^ | 127.86±5.24^b^ |

Note: Values marked with different lowercase letters indicate a significant difference (*P* < 0.05), whereas those marked with the same lowercase letters indicate no significant difference (*P* > 0.05).
